# Off‐target inhibition of NGLY1 by the polycaspase inhibitor Z‐VAD‐fmk induces cellular autophagy

**DOI:** 10.1111/febs.16345

**Published:** 2022-01-18

**Authors:** Sarah H. Needs, Martin D. Bootman, Jeff E. Grotzke, Holger B. Kramer, Sarah A. Allman

**Affiliations:** ^1^ 5488 School of Life, Health and Chemical Sciences The Open University Milton Keynes UK; ^2^ Reading School of Pharmacy University of Reading UK; ^3^ 12228 Yale University School of Medicine New Haven CT USA; ^4^ 47697 Department of Physiology, Anatomy and Genetics University of Oxford UK; ^5^ 47697 MRC London Institute of Medical Sciences UK; ^6^ Leicester School of Pharmacy De Montfort University Leicester UK

**Keywords:** autophagosome proteomics, autophagy, NGLY1, Peptide: *N*‐glycanase 1, Z‐VAD‐fmk

## Abstract

The polycaspase inhibitor Z‐VAD‐fmk acts as an inhibitor of peptide: *N*‐glycanase (NGLY1), an endoglycosidase which cleaves *N*‐linked glycans from glycoproteins exported from the endoplasmic reticulum (ER) during ER‐associated degradation (ERAD). Both pharmacological *N*‐glycanase inhibition by Z‐VAD‐fmk and siRNA‐mediated knockdown (KD) of NGLY1 induce GFP‐LC3‐positive puncta in HEK 293 cells. The activation of ER stress markers or induction of reactive oxygen species (ROS) is not observed under either condition. Moreover, Ca^2+^ handling is unaffected when observing release from intracellular stores. Under conditions of pharmacological NGLY1 inhibition or NGLY1 KD, upregulation of autophagosome formation without impairment of autophagic flux is observed. Enrichment of autophagosomes by immunoprecipitation (IP) and mass spectrometry‐based proteomic analysis reveals comparable autophagosomal protein content. Gene ontology analysis of proteins enriched in autophagosome IPs shows overrepresentation of factors involved in protein translation, localization and targeting, RNA degradation and protein complex disassembly. Upregulation of autophagy represents a cellular adaptation to NGLY1 inhibition or KD, and ATG13‐deficient mouse embryonic fibroblasts (MEFs) show reduced viability under these conditions. In contrast, treatment with pan‐caspase inhibitor, Q‐VD‐OPh, does not induce cellular autophagy. Therefore, experiments with Z‐VAD‐fmk are complicated by the effects of NGLY1 inhibition, including induction of autophagy, and Q‐VD‐OPh represents an alternative caspase inhibitor free from this limitation.

**Enzymes:**

Peptide:*N*‐glycanase1, Peptide‐*N*(4)‐(*N*‐acetyl‐beta‐glucosaminyl)asparagine amidase [**EC:3.5.1.52**].

## Graphical abstract

Inhibition of NGLY1 by polycaspase inhibitor Z‐VAD‐fmk in HEK293 cells leads to induction of autophagy and increase in autophagosome formation rather than disruption of autophagic flux. Similarly, autophagy induction is observed with NGLY1 siRNA knockdown. Immunoprecipitation and proteomic analysis reveal comparable autophagosomal protein content in both Z‐VAD‐fmk treatment and NGLY1 knockdown. The alternative broad‐spectrum caspase inhibitor Q‐VD‐OPh does not inhibit NGLY1, and no induction of autophagy is observed.

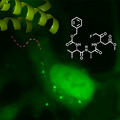

**Abbreviations**
3‐MA: 3‐methyladenine
ATG13: autophagy‐related protein 13
Boc‐D‐fmk: *tert*‐butyloxycarbonyl‐Asp(OMe) fluoromethyl ketone
ddVenus assay: deglycosylation‐dependent Venus assay
DMEM: Dulbecco's modified Eagle medium
ENGase: cytosolic endo‐beta‐N‐acetylglucosaminidase
ERAD: endoplasmic reticulum‐associated degradation
FASP: filter‐aided sample preparation
FDR: false discovery rate
Fura‐2 AM: Fura‐2 pentakis(acetoxymethyl) ester
GFP: green fluorescent protein
GlcNAc: *N*‐acetyl glucosamine
GO: gene ontology
HBSS: Hank’s balanced salt solution
HCA: hierarchical clustering analysis
IP: immunoprecipitation
KD: small‐interfering RNA‐mediated knockdown
LC3: autophagy‐related protein LC3 B
LC‐MS/MS: liquid chromatography‐tandem mass spectrometry
LFQ: label‐free quantification
MEF: mouse embryonic fibroblast
MTT: methylthiazolyl diphenyl tetrazolium bromide
NFE2L1: nuclear factor, erythroid‐derived 2, like 1
NGLY1 (*Homo sapiens*), PNG1 (*Saccharomyces cerevisiae*): Peptide:*N*‐glycanase 1
qPCR: quantitative real‐time polymerase chain reaction
Q‐VD‐OPh: quinoline‐Val‐Asp‐2,6‐difluorophenoxymethyl ketone
ROS: reactive oxygen species
ThT: thioflavin T
Z‐IETD‐fmk: *N*‐benzyloxycarbonyl‐Ile‐Glu(OMe)‐Thr‐Asp(OMe) fluoromethyl ketone
Z‐VAD‐fmk: *N*‐benzyloxycarbonyl‐Val‐Ala‐Asp(OMe) fluoromethyl ketone

## Introduction

The peptide fluoromethyl ketone inhibitor Z‐VAD‐fmk has been applied extensively to the study of caspases and the central role of this important group of cysteine proteases in apoptosis. The widespread use of this inhibitor is likely due to its effective inhibition of a range of caspases and its extensive application for over more than 20 years in biological research. Utilization as a tool in cellular research continues to date, despite the recognition that Z‐VAD‐fmk also interacts with several off‐targets. These off‐targets include other cysteine proteases, such as the cathepsins [1] and calpains [2], but also the amidase peptide:*N‐*glycanase 1 (NGLY1) [3]. Additionally, it has been shown that both Z‐VAD‐fmk and Z‐IETD‐fmk, a related peptide fluoromethyl ketone (fmk) inhibitor, are capable of inhibiting rhinoviral proteinases and thereby reducing viral replication in cultured cells [4]. The selectivity profile of Z‐VAD‐fmk across human caspases has been characterized in detail [5]. Furthermore, quantitative comparisons of inhibition profiles between Z‐VAD‐fmk and Q‐VD‐OPh across caspases and cathepsins have shown that the latter represents a more potent and more selective second‐generation inhibitor [6, 7]. Despite this, Z‐VAD‐fmk is still in recent and current use in a large number of studies including for the induction of necroptosis [8, 9] or continued use for the inhibition of caspase‐mediated apoptosis [10]. While inhibition of cellular caspases leads to suppression of apoptosis, it has been shown that Z‐VAD‐fmk treatment can induce other forms of programmed cell death, such as autophagic cell death in L929 fibrosarcoma cells [11]. The authors showed that cellular autophagy, including autophagosome and autolysosome formation, was central in this pathway to cell death and implicated inactivated caspase 8 and increased ROS production in the process. Disparate cellular effects of different caspase inhibitors have also been documented in the literature. Investigations with the inhibitors Z‐VAD‐fmk, Boc‐D‐fmk and Q‐VD‐OPh in L929 cells revealed that the two fmk‐based inhibitors induced necroptosis, while Q‐VD‐OPh did not [12]. The investigators went on to show that autocrine production of TNF‐α was involved in this process and was mediated by the PKC‐MAPKs‐AP1 pathway. Furthermore, they demonstrated that the NF‐kB pathway exerts a protective function against necroptosis. Despite such disparate cellular outcomes for different caspase inhibitors, the precise mechanistic reasons for varying outcomes with different inhibitors are often unclear. The fmk pharmacophore has been implicated in potential undesired effects due to metabolic turnover into toxic fluoroacetate [13] which, following conversion to fluorocitrate, is capable of inactivating aconitase within the citric acid cycle [14]. It should be noted, however, that significant differences in selectivity between fmk‐based and alternative inhibitors itself may explain differences in engagement of cellular off‐targets.

The primary biochemical function of the known Z‐VAD‐fmk off‐target NGLY1 is the enzymatic removal of *N*‐linked glycans from glycoprotein substrates [15, 16], and its involvement in the endoplasmic reticulum‐associated degradation (ERAD) pathway has been clearly demonstrated [17, 18]. Furthermore, NGLY1 deficiency is a rare genetic disorder with a complex clinical presentation which often includes neurological symptoms and developmental delay [19, 20]. Recent findings have demonstrated novel cellular functions of NGLY1 in the regulation of aquaporins independent of its enzymatic activity [21] and *N*‐glycanase‐dependent sequence editing of NFE2L1 (also known as Nrf‐1), leading to the induction of gene expression of proteasomal subunits [22, 23, 24]. Recently, it has also been shown that knockdown (KD) of NGLY1 in K562 human myelogenous leukaemia cells increases sensitivity towards proteasome inhibition and induces complex changes in transcript and protein regulation [25]. Despite these important findings, there is still limited mechanistic information on the effects of NGLY1 inhibition or disruption in mammalian cells.

The aim of this study is to delineate to which extent the cellular effects observed upon treatment with Z‐VAD‐fmk are mediated by the inhibition of its off‐target NGLY1.

## Results

### Pan‐caspase inhibitor Z‐VAD‐fmk, but not Q‐VD‐OPh, inhibits *N*‐glycanase activity of NGLY1

**Fig. 1:**
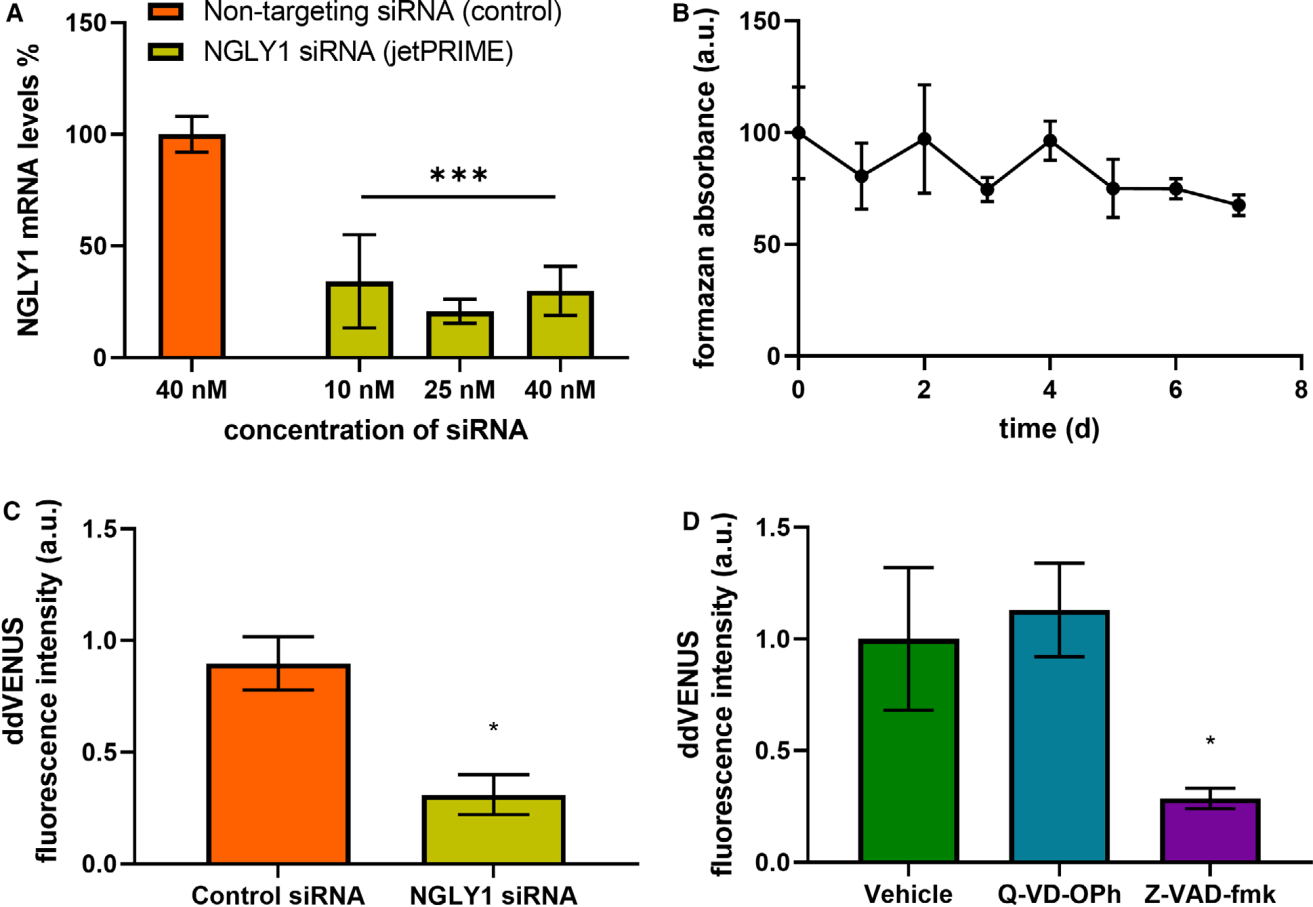
*N*‐glycanase siRNA knockdown and Z‐VAD‐fmk treatment reduce the amount of cellular deglycosylation. (A) Transfection of NGLY1 siRNA in HEK 293 cells results in significant reduction of NGLY1 mRNA levels (compared with nontargeting control). Optimization of transfection of NGLY1 siRNA in HEK 293 cells. Data represent the percentage mRNA knockdown at different concentrations of targeting siRNA (10, 25 and 40 nM using jetPRIME™ as a transfection reagent) compared to a nontargeting control siRNA. Two‐way ANOVA, Dunnett’s *post hoc* compared to nontargeting siRNA. **P < 0.01, ***P < 0.001, error bars ± SEM, *n* = 3. (B) Transfection of NGLY1 siRNA in HEK 293 cells does not cause significant decrease in cellular viability. MTT cell viability assay. HEK 293 cells transfected with 25 nm NGLY1 siRNA over 7 days. Data normalized to nontargeting control of the same time and Day 0 represented as 100%. One‐way ANOVA, followed by a Dunnett’s *post hoc* test against the vehicle. HEK 293 cells were treated with either Q‐VD‐OPh or Z‐VAD‐fmk (50 µm) for 24 h (C) or transfected with NGLY1 siRNA or nontargeting siRNA (25 nm) with jetPRIME^®^ for 3 days (D) followed by transfection with ddVENUS construct with jetPEI^®^. After 72 h, cells were treated with MG132 (8 µm) for 6 h and fluorescence intensity was analysed by flow cytometry. The median ddVENUS fluorescence was calculated and normalized to the vehicle. *n* = 3, error bars indicate ± SEM. * indicates *P* < 0.05.

We were interested in investigating the cellular effects of inhibition of NGLY1 by Z‐VAD‐fmk, a known off‐target for this pan‐caspase inhibitor. siRNA‐mediated gene knockdown (KD) in HEK 293 cells leads to a significant reduction in NGLY1 transcript levels (Fig. 1A) while cell viability remains unaffected (Fig. 1B). In order to assess remaining enzymatic *N*‐glycanase activity in NGLY1 KD cells, a deglycosylation‐dependent Venus (ddVenus) assay [17] was employed. This demonstrated a significant reduction of *N*‐glycanase activity in NGLY1 KD cells by 68% at 72 h post‐transfection (Fig. 1C). A similar significant reduction of 72% of *N*‐glycanase activity was observed with the same ddVenus assay following treatment of HEK 293 cells with 50 µm Z‐VAD‐fmk for 24 h (Fig. 1D). It should be noted that no detrimental effects on cellular viability were observed at or below concentrations of 100 µm (Fig. S1). Conversely, HEK 293 cells treated with 50 µm Q‐VD‐OPh for the same time period showed no significant alteration of fluorescence intensity in the ddVenus assay, indicating that cellular *N*‐glycanase activity remained unaffected (Fig. 1D).

### Z‐VAD‐fmk treatment induces cellular autophagy via NGLY1 inhibition in HEK 293 cells

**Fig. 2:**
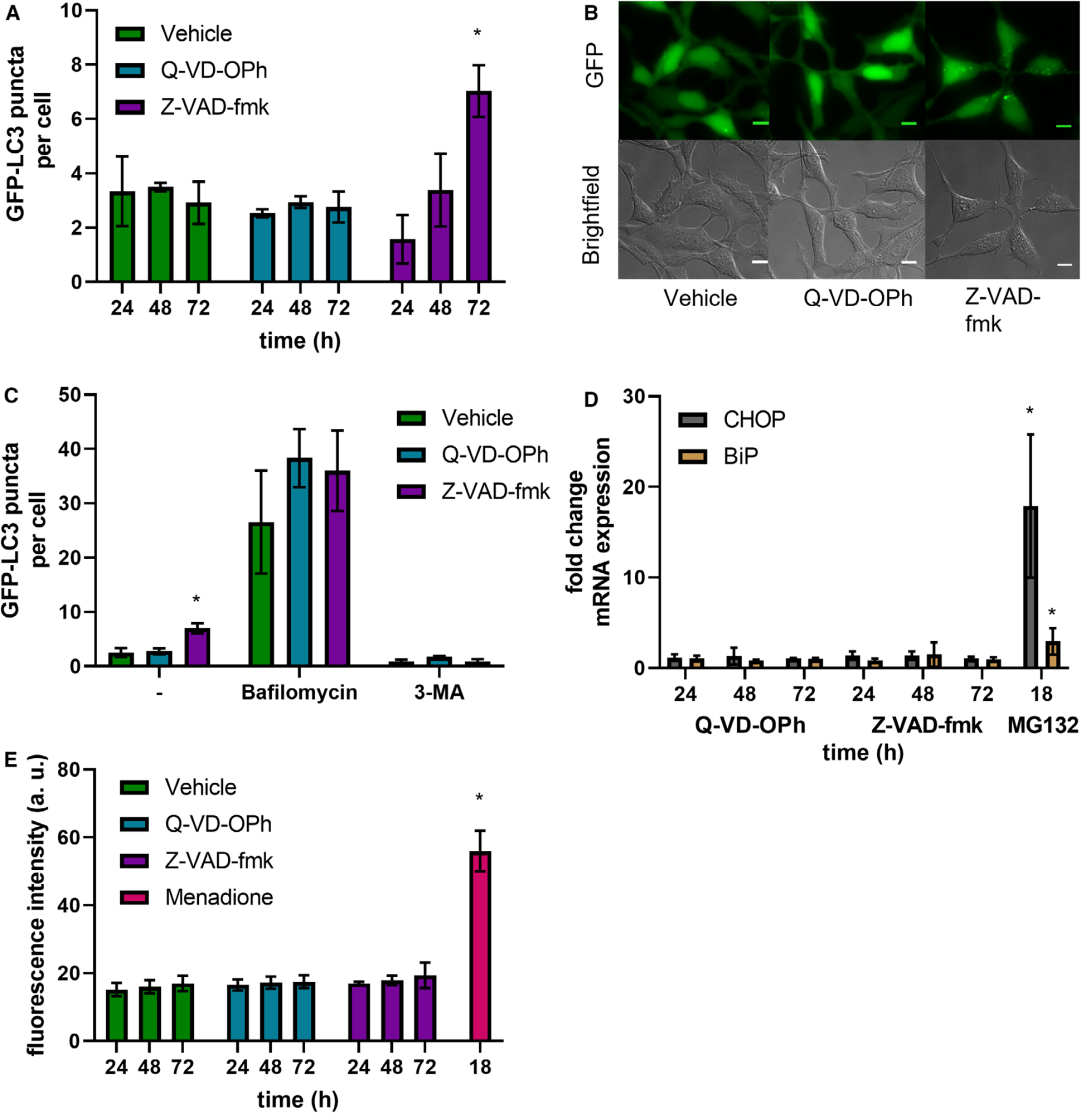
Z‐VAD‐fmk, but not Q‐VD‐OPh, induces autophagy. (A) GFP‐LC3 puncta per cell after treatment with Z‐VAD‐fmk or Q‐VD‐OPh (50 µm, 24–72 h). (B) Representative images of treatment of Z‐VAD‐fmk or Q‐VD‐OPh after 72 h. Scale bar indicates 10 µm. (C) GFP‐LC3 puncta per cell after 72‐h treatment with Z‐VAD‐fmk or Q‐VD‐OPh followed by bafilomycin A1 treatment (1 h, 100 nm) or 3‐MA (1 h, 5 mm). Two‐way ANOVA, Tukey’s *post hoc*. (D) rt‐qPCR analysis of CHOP and BiP in HEK 293 cells treated with Z‐VAD‐fmk or Q‐VD‐OPh (50 µm, 24–72 h). mRNA levels normalized to GAPDH and a vehicle control using ΔΔCt method. Two‐way ANOVA, Tukey’s *post hoc* within each mRNA target. (E) HEK 293 cells treated with Z‐VAD‐fmk or Q‐VD‐OPh (50 µm, 24–72 h). Cells were stained with ROS Brite 570 (5 µm, 0.5 h). Two‐way ANOVA, Tukey’s *post hoc*, error bars ± SEM, *n* = 3, *P < 0.05.

Given the different effects of Z‐VAD‐fmk and Q‐VD‐OPh with respect to inhibition of cellular *N*‐glycanase activity, we were interested in comparing the cellular effects of the two inhibitors more broadly. Previous reports have implicated an involvement of autophagy in necrotic cell death which can be observed in some cell lines treated with Z‐VAD‐fmk [11, 12, 26]. Treatment of stably transfected GFP‐LC3 HEK 293 cells with Z‐VAD‐fmk (50 µm), Q‐VD‐OPh (50 µm) or vehicle control for 24, 48 or 72 h showed a significant increase in the number of GFP‐LC3 puncta per cell at the 72‐h time point in the Z‐VAD‐fmk‐treated cells (Fig. 2A,B). A control experiment to assess any potential disruption of autophagic flux was also carried out (Fig. 2C).

### Lack of ER stress or Redox imbalance in cells subjected to Z‐VAD‐fmk, Q‐VD‐OPh or NGLY1 KD

**Fig. 3:**
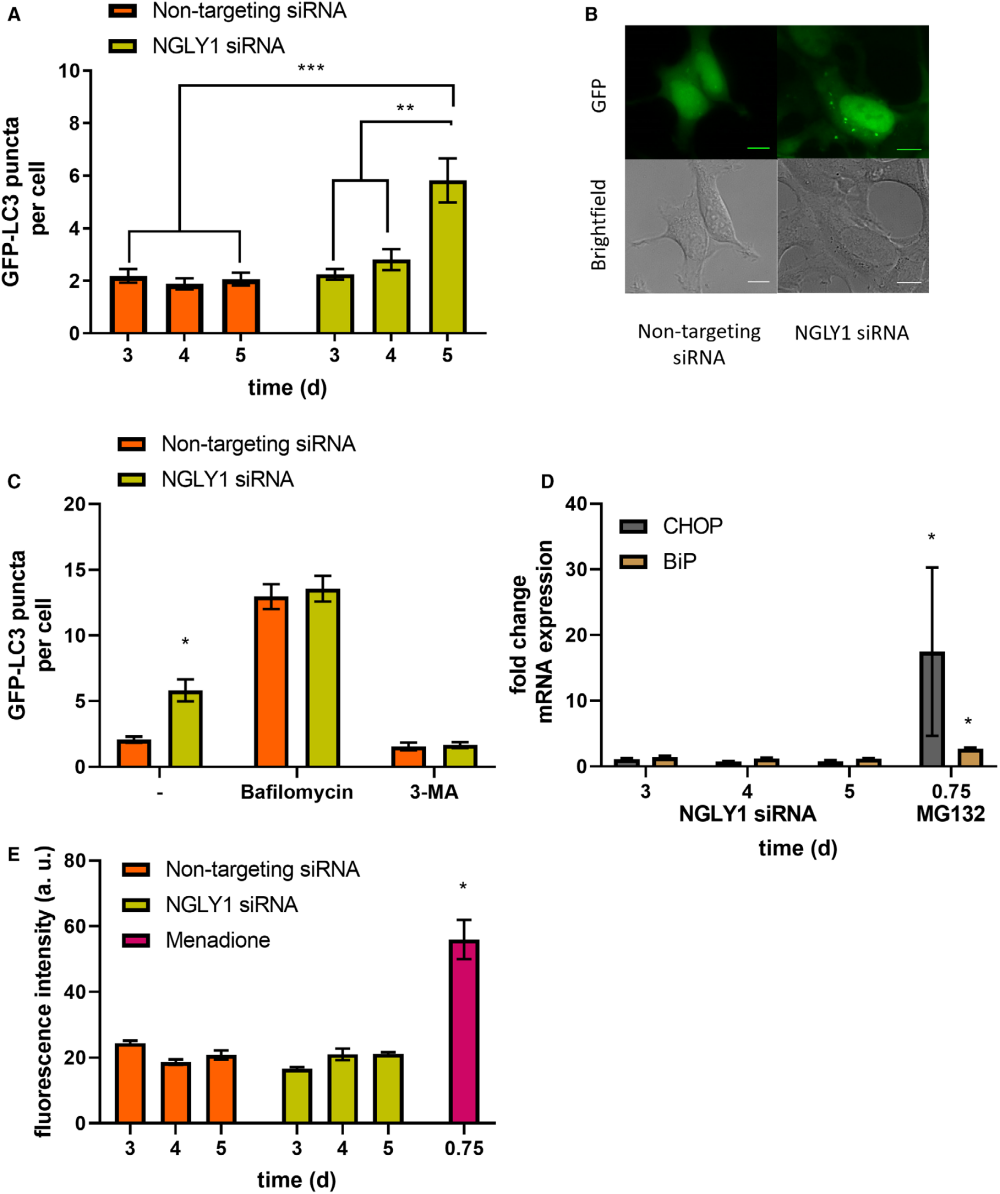
siRNA knockdown of *N*‐glycanase induces autophagy. (A) GFP‐LC3 puncta are increased after 5 days post‐transfection with NGLY1 siRNA (25 nm). Quantitation of the average GFP‐LC3 puncta per cell, minimum of three images per coverslip with three biological replicates. Error bars indicate ± SEM, **P < 0.01, ***P < 0.001, two‐way ANOVA, Tukey’s *post hoc*. (B) Representative images of HEK 293 cells transfected with NGLY1 siRNA or nontargeting siRNA after 5 days. Scale bar indicates 10 µm. (C) GFP‐LC3 puncta per cell after transfection with NGLY1 siRNA or nontargeting siRNA (25 nm, 5 days) with bafilomycin A1 (100 nm, 1 h) or 3‐MA (5 mm, 1 h). (D) rt‐qPCR analysis of CHOP and BiP in HEK 293 cells transfected with NGLY1 siRNA (25 nm, 3–5 days), mRNA levels normalized to GAPDH and a nontargeting siRNA control using ΔΔ*C*_t_ method. Two‐way ANOVA, Tukey’s *post hoc* within each mRNA target. (E) HEK 293 cells transfected with NGLY1 siRNA or nontargeting siRNA (25 nm, 3–5 days) and incubated with ROS Brite™ 570 (5 µm, 0.5 h). Two‐way ANOVA, Tukey’s *post hoc*. *P < 0.05, Error bars ± SEM, *n* = 3.

As autophagy activation has been reported following ER stress, potential induction of the endoplasmic reticulum (ER) stress markers C/EBP homologous protein (CHOP) and binding‐immunoglobulin protein (BiP) was monitored using quantitative real‐time polymerase chain reaction (qPCR) (Fig. 2D). No induction of ER stress markers was observed for either Z‐VAD‐fmk or Q‐VD‐OPh treatment (each at 50 µm) at the time points tested (24, 48 and 72 h), while the positive control treatment with proteasome inhibitor MG132 (5 µm, 18 h) showed robust induction of both ER stress markers. We also explored the possible effects on cellular redox homeostasis and production of reactive oxygen species (ROS). To this end, we stained HEK 293 cells with ROS Brite™ 570 following treatment with either vehicle control, Z‐VAD‐fmk or Q‐VD‐OPh (50 µm each). This showed that across treatment periods of 24–72 h, no significant induction of cellular ROS could be observed. As a positive control, menadione treatment (5 µm, 18 h) showed a significant increase in the measured ROS Brite™ fluorescence (Fig. 2E). In order to confirm whether the induction of autophagosome formation, as shown by induction of LC3 puncta, can be explained by the inhibition of NGLY1, we carried out a comparable experiment with siRNA‐mediated NGLY1 KD (Fig. 3A,B). A significant increase in GFP‐LC3 puncta was observed at 5 days post‐transfection when comparing NGLY1 KD to the nontargeting siRNA control. A further control experiment to assay for disruption of autophagic flux following NGLY1 KD was also conducted (Fig. 3C). As with the inhibitor treatment experiments (Fig. 2D,E), we investigated induction of ER stress markers CHOP and BiP, and cellular ROS in this system. We found no significant differences between nontargeting siRNA control and NGLY1 KD cells (Fig. 3D,E) and thereby no evidence for induction of ER stress or disruption of cellular redox balance.

### Induction of autophagosome formation rather than disruption of autophagic flux is responsible for NGLY1‐mediated increase in cellular autophagy

In order to assess whether the observed induction of autophagosome formation in Z‐VAD‐fmk treatment was caused by a disruption of autophagic flux or due to genuine upregulation of cellular autophagosome formation, two control experiments were carried out. Treatment of stably transfected GFP‐LC3 HEK 293 cells with Z‐VAD‐fmk or Q‐VD‐OPh for 72 h was followed by inhibition with bafilomycin or 3‐methyladenine (3‐MA) for 1 h (Fig. 2C). This showed no evidence for disruption of cellular autophagic flux. Similarly, treatment of stably transfected GFP‐LC3 HEK 293 cells with bafilomycin or 3‐methyladenine (3‐MA) following transfection with either nontargeting control siRNA or NGLY1 KD for 5 days was investigated (Fig. 3C). While the previously observed induction of autophagosome formation in NGLY1 KD was recapitulated, there was no significant difference between control siRNA and NGLY1 KD for both bafilomycin and 3‐MA treatment (Fig. 3C). This indicates that cellular autophagic flux is not affected in NGLY1 KD and a genuine increase in autophagosome formation is responsible for the observed elevation of GFP‐LC3 puncta per cell.

### Cellular viability upon Z‐VAD‐fmk treatment is reduced in ATG13 KO compared with WT MEF cells

We also performed MTT assays of wild‐type mouse embryonic fibroblasts (WT MEFs) and ATG13 knock‐out (KO) MEF cells treated with either Z‐VAD‐fmk or Q‐VD‐OPh. ATG13 is essential for cellular autophagy, and KO cells have strongly diminished ability to induce autophagosome formation [27]. Having confirmed ATG13 deficiency in the KO cells by determining transcript levels by qPCR (Fig. S2a), we compared the effects of inhibitor treatment of WT and ATG13 KO MEF cells (Fig. S2b,c). Upon Z‐VAD‐fmk treatment, a significant difference in formazan absorbance was observed at inhibitor concentrations of 100 and 200 µm (Fig. S2b). In contrast, no significant difference between WT and ATG13 KO MEFs was observed for Q‐VD‐OPh at all concentrations tested (Fig. S2c). As a further control experiment, Z‐VAD‐fmk treatment following siRNA‐mediated KD of ATG13 in WT MEF cells was compared to nontargeting siRNA‐transfected cells (Fig. S2d). This was performed to ensure that experiments with ATG13 KO cells were not confounded by cellular adaptation effects. The corresponding MTT assay showed a significant decrease in formazan absorbance in ATG13 KD compared with the nontargeting siRNA control.

### Transient increase in Thioflavin T fluorescence is observed after treatment with Z‐VAD‐fmk but not Q‐VD‐OPh

To examine whether protein aggregation was observed on treatment with Z‐VAD‐fmk or Q‐VD‐OPh, cells were visualized with thioflavin T (ThT) following inhibitor treatment for 24, 48 and 72 h and changes in fluorescence measured by flow cytometry. Flow cytometric analysis of treated cells indicated a transient increase in ThT fluorescence at 48 h upon treatment with Z‐VAD‐fmk and no significant change in fluorescence upon treatment with Q‐VD‐OPh (Fig. S3a). This observation aligns with our data showing significant induction of autophagy at 72 h post‐Z‐VAD‐fmk treatment.

### Intracellular Ca^2+^ handling is unaffected by treatment with Z‐VAD‐fmk, Q‐VD‐OPh or NGLY1 KD

In order to assess whether intracellular Ca^2+^ signalling is affected by Z‐VAD‐fmk or Q‐VD‐OPh inhibitor treatment, we studied whether release of Ca^2+^ from intracellular stores is affected under these conditions. HEK 293 cells were loaded with Fura‐2 AM dye (1 µm) following inhibitor treatment (vehicle control, Z‐VAD‐fmk or Q‐VD‐OPh) and then Ca^2+^ release stimulated with thapsigargin (1 µm). Fluorescence imaging was carried out with excitation at 340 and 380 nm wavelengths allowing ratiometric determination of free intracellular Ca^2+^ concentrations. These measurements indicated no significant difference in Ca^2+^ release from intracellular stores following inhibitor treatments with either Z‐VAD‐fmk or Q‐VD‐OPh (50 µm each, 24–72 h) relative to vehicle control (Fig. S4a–f). Comparable experiments were also conducted following NGLY1 KD or treatment with nontargeting control siRNA. Again, no significant difference in Ca^2+^ release from intracellular stores was observed at any of the time points (3–5 days) tested (Fig. S5a–e).

### Autophagosome immunoprecipitation and mass spectrometry‐based proteomics identifies autophagosomal protein content following Z‐VAD‐fmk treatment or NGLY1 KD

In order to investigate autophagosomal proteins at the molecular level, we performed autophagosome proteomic analysis of stably transfected GFP‐LC3 HEK 293 cells which had undergone either Z‐VAD‐fmk treatment or NGLY1 KD. Autophagosomes were enriched by centrifugation followed by anti‐GFP immunoprecipitation (IP), and corresponding negative control IPs were carried out in parallel. All experiments were conducted as biological triplicates. Sample processing and analysis was carried out by trypsin digestion and liquid chromatography‐tandem mass spectrometry (LC‐MS/MS). Raw data were then processed and analysed in MaxQuant [28] using the label‐free quantification (LFQ) algorithm [29] and statistical analysis and data visualization in Perseus [30]. After removal of known contaminant proteins, this led to the identification 1011 protein hits at a false discovery rate (FDR) of 1%. Filtering to obtain only proteins which were quantified in at least two out of three replicates per experimental group retained 915 protein hits.

**Fig. 4:**
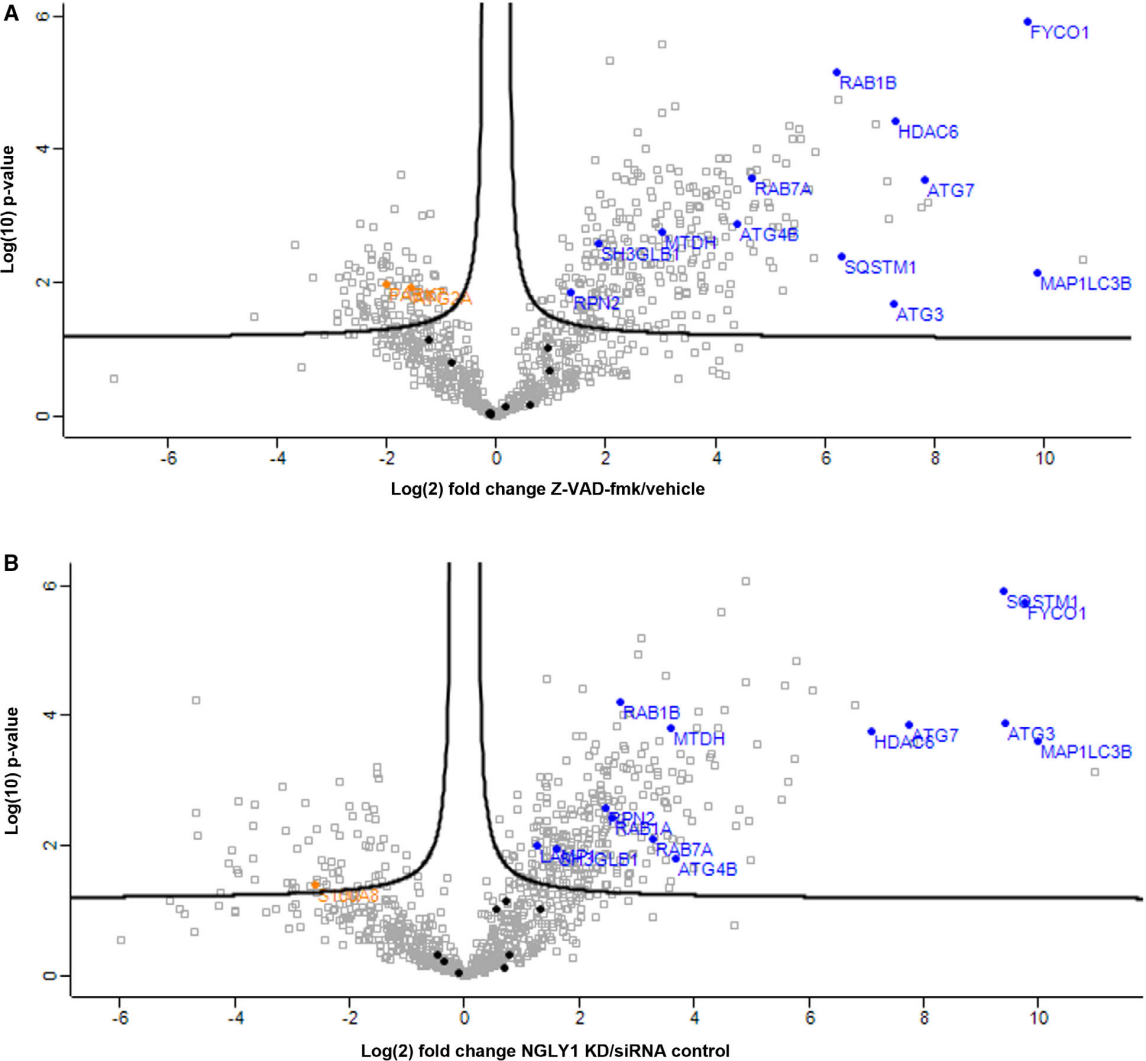
Label‐free proteomic analysis of autophagosomes isolated from HEK 293 cells treated with Z‐VAD‐fmk or NGLY1 KD. Volcano plots identifying significantly enriched and depleted proteins (FDR: 0.05, s0: 0.1) in autophagosomes enriched following (A) Z‐VAD‐fmk treatment or (B) NGLY1 KD relative to negative control IPs. Autophagy‐related proteins are highlighted in blue (significantly enriched), black (not significantly altered) or orange (significantly depleted).

**Fig. 5:**
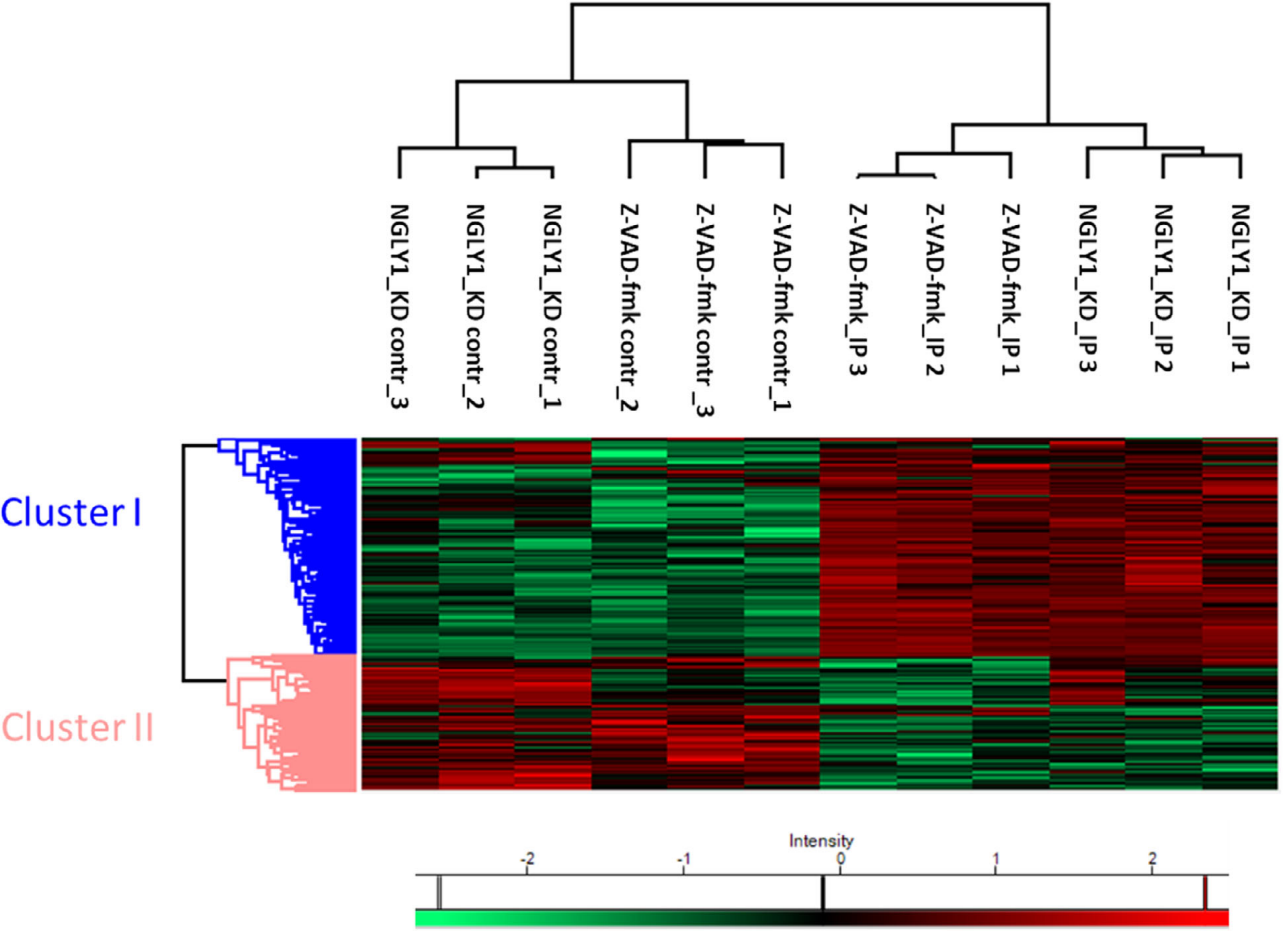
Label‐free quantitative proteomics identifies significantly altered protein hits in autophagosome IPs following Z‐VAD‐fmk treatment or NGLY1 KD. Heatmap and hierarchical clustering analysis (HCA) of significantly changing (one‐way ANOVA, FDR: 0.05) protein hits quantified following autophagosome IP or negative control IP and LC‐MS/MS analysis. Log(2)‐transformed label‐free quantification (LFQ) intensities after Z‐scoring and filtering to retain only ANOVA significant hits. Two clusters represent protein hits enriched in in autophagosome IPs (cluster I) and protein hits enriched negative control IPs (cluster II).

The label‐free quantitative proteomic data show enrichment of a subset of autophagy‐related proteins (Table 1) in autophagosomes isolated from Z‐VAD‐fmk treatment or NGLY1 KD, relative to the corresponding control IPs. The observation of multiple autophagy‐related proteins validates the experimental workflow and successful enrichment of autophagosomes. Strong enrichment is also shown in volcano plots comparing the GFP IPs to their respective negative control IPs for Z‐VAD‐fmk treatment or NGLY1 KD (Fig. 4A,B). Among the notable proteins which were found to be significantly enriched in the autophagosome IPs in both inhibitor treatment and NGLY1 KD were the bait protein MAP1LC3B, several known interactors thereof (MAP1A, MAP1B, FYCO1, SQSTM1), several autophagy‐related proteins (ATG3, ATG4B, ATG7), other known autophagosomal proteins (RAB1B, RAB7A, SH3GLB1) as well as known regulators of autophagy (HDAC6, HDAC10, MTDH). Direct comparison of the two autophagosome IPs from inhibitor treatment and NGLY1 siRNA KD was also performed by volcano plot (Fig. S6b) indicating no significant changes between the two IPs. A large degree of similarity between the two IPs is also evident in the heatmap with hierarchical clustering analysis (HCA) (Fig. 5). Filtering for ANOVA significant (FDR 0.05) proteins prior to HCA yields 369 significantly altered protein hits. These are subdivided into two clusters of proteins enriched in the autophagosome IPs (cluster I, 228 protein hits) and proteins enriched in the negative control IPs (cluster II, 141 protein hits). Investigation of the protein hits represented in clusters I and II by gene ontology (GO) analysis highlights biological processes (GOBP), cellular compartments (GOCC) and molecular functions (GOMF) which are overrepresented in the respective cluster (Tables S1 and S2). For proteins enriched in the autophagosome IPs, the GOBP terms which are overrepresented include protein translation (translation, translational elongation, translational termination, translational initiation), protein localization and targeting (SRP‐dependent cotranslational protein targeting to membrane, protein targeting to ER, establishment of protein localization to organelle, establishment of protein localization in endoplasmic reticulum, protein targeting to membrane, cotranslational protein targeting to membrane, establishment of localization), RNA degradation (nuclear‐transcribed mRNA catabolic process – nonsense‐mediated decay, mRNA catabolic process, RNA catabolic process nuclear‐transcribed mRNA catabolic process) and protein complex disassembly (protein complex disassembly, cellular component disassembly, macromolecular complex disassembly, cellular component disassembly at cellular level, cellular macromolecular complex disassembly). GOCC and GOMF terms overrepresented in the autophagosome IPs are largely focused on ribosomes (GOCC: cytosolic large ribosomal subunit, large ribosomal subunit; GOMF: structural constituent of ribosome). These results confirm successful enrichment of autophagosomes following Z‐VAD‐fmk treatment or NGLY1 KD, identification of similar autophagosomal protein composition for both conditions and the identification of autophagosomal cargo proteins and the corresponding biological processes represented.

**Table 1:** Enrichment of autophagy‐related proteins in autophagosome IPs following Z‐VAD‐fmk or NGLY1 KD. Tabulated autophagy‐related proteins shown with number of unique peptides identified, percentage sequence coverage, andromeda score and MS/MS count. Only protein hits with ≥ 2 tryptic peptides/protein are displayed. Significant enrichment: ‘++’ significantly enriched in both autophagosome IPs; ‘+’ significantly enriched in one autophagosome IP (either following Z‐VAD‐fmk treatment or NGLY1 KD); ‘ ’ no significant enrichment in either autophagosome IP.

Significant enrichment	Protein names	Majority protein IDs	Gene names	Unique peptides	Sequence coverage [%]	Andromeda score	MS/MS count
++	Ubiquitin‐like‐conjugating enzyme ATG3	Q9NT62	ATG3	15	38.9	323.31	159
++	Ubiquitin‐like modifier‐activating enzyme ATG7	O95352	ATG7	26	48.2	323.31	242
++	FYVE and coiled‐coil domain‐containing protein 1	Q9BQS8	FYCO1	79	57.8	323.31	606
++	Histone deacetylase 6	Q9UBN7	HDAC6	20	25.5	323.31	142
++	Microtubule‐associated protein 1A	P78559	MAP1A	54	30.9	323.31	206
++	Microtubule‐associated protein 1B	P46821	MAP1B	160	69.7	323.31	1498
++	Sequestosome‐1	Q13501	SQSTM1	19	63.9	323.31	160
++	Ras‐related protein Rab‐1B	Q9H0U4	RAB1B	4	48.8	111.55	43
++	Microtubule‐associated proteins 1A/1B light chain 3B	Q9GZQ8; A6NCE7	MAP1LC3B	7	31.2	44.931	232
+	Cysteine protease ATG4B	Q9Y4P1	ATG4B	6	23.7	44.849	43
	Next to BRCA1 gene 1 protein	Q14596	NBR1	5	8.4	29.383	12
++	Histone deacetylase 10	Q969S8	HDAC10	11	28.3	25.14	70
++	Ras‐related protein Rab‐7a	P51149	RAB7A	7	42	22.24	39
	Ubiquilin‐2	Q9UHD9	UBQLN2	4	11.1	15.824	8
+	Endophilin‐B1	Q9Y371	SH3GLB1	2	7.4	11.298	14
	Lysosome‐associated membrane glycoprotein 1	P11279	LAMP1	3	8.6	9.1387	7
++	Protein LYRIC	Q86UE4	MTDH	6	13.2	8.8476	20
+	Ras‐related protein Rab‐1A	P62820	RAB1A	2	35.6	8.2661	15
	Microtubule‐associated protein 1S	Q66K74	MAP1S	3	4.9	6.7001	4
	Protein deglycase DJ‐1	Q99497	PARK7	2	14.3	4.4783	4
	Gamma‐aminobutyric acid receptor‐associated protein‐like 2	P60520	GABARAPL2	2	16.2	1.7581	3
	Vesicle‐associated membrane protein 8	Q9BV40	VAMP8	2	19	1.6776	2

## Discussion

Interest in NGLY1 has been focused on its central role in the rare genetic disorder NGLY1 deficiency and in its importance towards cellular glycoprotein turnover. NGLY1 is also a known off‐target of the pan‐caspase inhibitor Z‐VAD‐fmk, and our results show that siRNA‐mediated knockdown of NGLY1 achieves a comparable reduction in cellular peptide:*N*‐glycanase activity as that observed with Z‐VAD‐fmk treatment (24 h, 50 µm) (Fig. 1C,D). In contrast, treatment with the alternative caspase inhibitor Q‐VD‐OPh does not affect cellular peptide:*N*‐glycanase activity in HEK 293 cells (Fig. 1D), a result which is in agreement with previous studies on *S. cerevisiae* PNG1 [3]. A broader investigation of cellular responses to inhibitor treatment showed no evidence of induction of ER stress markers or ROS production upon Z‐VAD‐fmk or Q‐VD‐OPh treatment in HEK 293 cells (Fig. 2D,E). However, induction of cellular autophagosome formation (determined by quantification of GFP‐LC3 puncta) was observed following treatment with Z‐VAD‐fmk at 72 h (50 µm), but not with Q‐VD‐OPh treatment in stably transfected GFP‐LC3 HEK 293 cells (Fig. 2A,B). Induction of autophagosome formation was also observed with siRNA‐mediated KD of NGLY1 at 5 days post‐transfection, but not with the scrambled control siRNA (Fig. 3A,B). These results strongly suggest that induction of autophagosomes in Z‐VAD‐fmk treatment is related to NGLY1 inhibition and not to caspase inhibition or other potential off‐targets.

As for the inhibitor treatment experiments, ER stress markers or ROS production was determined and found not to be altered in NGLY1 KD relative to control (Fig. 3D,E). This result agrees with previous studies carried out on NGLY1 KD in *Drosophila melanogaster* [31] and in NGLY1 knock‐out (KO) rats [32] where no evidence of induction of ER stress was found. In addition, the rat model showed evidence for the accumulation of polyubiquitinated proteins in neurons from five‐week‐old NGLY1 KO rats [32]. The study in the *Drosophila* model also showed evidence for cnc (fly ortholog of NFE2L1) dysfunction by transcriptome analysis among downregulated genes (such as those encoding for proteasomal subunits) and an upregulation of the heat‐shock response [31]. Similar downregulation at the transcriptional level of proteasomal subunits was also observed following NGLY1 KD in human K562 cells and was interpreted as related to reduced post‐translational processing of NFE2L1 to the mature transcription factor by NGLY1 [25]. Consistent with these observations, it was found that NGLY1 disruption sensitizes towards proteasome inhibition by bortezomib in human cells [25] and *Drosophila* larvae [33]. The NGLY1 KO mouse model showed the importance of the genetic background towards embryonic lethality and demonstrated that concomitant KO of the ENGase gene partially rescued embryonic lethality in C57BL/6 mice [34]. It has been suggested that this observation may be explained by a detrimental effect of *N*‐GlcNAc‐modified proteins formed by ENGase‐mediated processing of *N*‐linked glycoproteins in the absence of functional NGLY1 [35]. In our study, we observe a transient increase in ThT fluorescence at 48 h (Fig. S3a) followed by a significant induction of autophagy at 72 h post‐Z‐VAD‐fmk treatment, suggesting the formation then clearance of aggregated material. Although we did not observe the same pattern upon NGLY1 KD (Fig. S3b), we suspect that the time course of pharmacological NGLY1 inhibition is difficult to mimic in the KD system. It is notable that similar experiments with K562 NGLY1 KD cells mirror these observations and also showed a trend towards increased protein aggregation, rather than a significant difference [25].

It is noteworthy that an induction of autophagy upon Z‐VAD‐fmk treatment has been observed previously in experiments with L929 cells where autophagic cell death is induced [11, 12, 26] and patient‐derived tenofibroblasts where autophagy induction is observed in the presence or absence of hydrogen peroxide cotreatment [36]. This suggests that Z‐VAD‐fmk‐mediated autophagy induction is not restricted to a narrow range of cells or cell lines. However, to the best of our knowledge, it has not been demonstrated before that this cellular effect is mediated by inhibition of NGLY1. In order to pinpoint the origin of the increase in levels of cellular autophagosomes, we compared blockage of lysosomal degradation with bafilomycin or inhibition of autophagosome biogenesis using 3‐methyladenine (3‐MA) for each vehicle, Z‐VAD‐fmk or Q‐VD‐OPh treatments at the 72‐h timepoint (Fig. 2C). The observed increases (bafilomycin) and decreases (3‐MA) in autophagosome content for all three conditions indicate that autophagic flux is not impaired in this cell model. This strongly suggests that the observed increased number of GFP‐LC3 puncta is the result of genuine induction of autophagosome formation and not a defect of autophagic flux. Importantly, corresponding experiments were also carried out with control siRNA and NGLY1 KD cells at 5 days post‐transfection, similarly showing no evidence of impairment in autophagic flux in cells deficient in NGLY1 (Fig. 3C). While an increase in autophagosome formation is a consequence of both Z‐VAD‐fmk treatment and NGLY1 KD, autophagy also represents an adaptation to reduced cellular *N*‐glycanase activity. Investigations using ATG13 KO MEF cells show that these ATG13‐deficient cells (Fig. S2a) are less tolerant towards treatment with Z‐VAD‐fmk. Cellular viability upon treatment with Z‐VAD‐fmk, as assessed by MTT reduction, was significantly reduced in ATG13 KO MEF cells when compared to control WT MEF cells (Fig. S2b). In contrast to this, no such difference between ATG13 KO and WT MEF cells could be observed for treatment with Q‐VD‐OPh across the concentration range of 0–200 µm (Fig. S2c). It is also noted that comparable reduction in cell viability is observed upon treatment of WT MEF cells with ATG13 siRNA (Fig. S2d), and as such, this effect is unlikely to be a result of adaptations in the ATG13 KO cell line. It has been shown previously that resveratrol‐induced autophagy, which is triggered via a noncanonical pathway, involves inositol triphosphate receptors and cytosolic Ca^2+^ in HEK293 cells [37]. Similar observations have also been made previously in the context of resveratrol‐induced autophagic cell death in A549 cells [38]. Therefore, we decided to investigate whether alterations in intracellular Ca^2+^ signalling are observed in Z‐VAD‐fmk or Q‐VD‐OPh inhibitor treatment or following NGLY1 KD. A further motivation for investigating cellular Ca^2+^ signalling was the observation that NGLY1 deficiency leads to impairment in mitochondrial function [24, 39] which could conceivably contribute to alterations in intracellular Ca^2+^ handling. However, our results (Figs S3a–f and S4a–e) indicate that neither treatment with Z‐VAD‐fmk or Q‐VD‐OPh nor NGLY1 KD significantly affect release of Ca^2+^ from intracellular stores by thapsigargin stimulation.

We carried out autophagosome enrichment by a two‐step process of centrifugation followed by IP of GFP‐LC3, in order to investigate autophagosomal protein content. LC3 specifically associates with autophagosomes following post‐translational processing to LC3‐II and represents the primary autophagosomal marker [40]. The enriched autophagosomes were then processed by lysis, protein denaturation and trypsin digestion for LC‐MS/MS and quantitative label‐free proteomic analysis (Fig. S6a). By conducting and processing negative control precipitations in parallel utilizing agarose beads, we assessed enrichment relative to control in order to identify autophagosome‐associated proteins and autophagosomal cargo. These experiments were carried out for both Z‐VAD‐fmk treatment and NGLY1 KD and were conducted in biological triplicates. Following data analysis and processing using previously established methods, enrichment of proteins was visualized using volcano plots (Fig. 4A,B). On the plots, 22 autophagy‐related proteins (Table 1) which were quantified in the analysis were highlighted and 14 of these were found to be significantly enriched (FDR: 0.05, s0: 0.1) following Z‐VAD‐fmk treatment and 13 significantly enriched in autophagosome IPs after NGLY1 KD (Table 1, Fig. 4A,B). Importantly, this included the bait protein LC3 and the known LC3‐interacting proteins MAP1A [41], MAP1B [41, 42], FYCO1 [43] and SQSTM1 [44] all of which were significant and highly enriched. Significant enrichment in both IP experiments was also observed for the autophagy proteins ATG3 and ATG7. Similarly, the regulator of autophagosome maturation HDAC6 [45], the positive autophagy regulator HDAC10 [46] and autophagy inducer MTDH [47] were found to be enriched and significant in both experiments. Direct comparison of the two autophagosome IPs following Z‐VAD‐fmk treatment or NGLY1 KD by volcano plot showed that there were no significantly altered protein hits between these conditions (FDR: 0.05, s0: 0.1), suggesting that both triggers of autophagy lead to comparable cellular autophagic responses and autophagosomal protein cargo (Fig. S6b). The entire proteomic data set was also displayed as a heatmap of protein abundance, filtered for ANOVA significant protein hits (FDR 0.05) and analysed by hierarchical clustering analysis (HCA) (Fig. 5). This approach further confirmed the similarity of proteins significantly enriched in the autophagosome IP experiments (cluster I, representing 228 protein hits). Gene Ontology (GO) analysis for biological process terms showed enrichment of protein translation, protein localization and targeting, mRNA degradation and protein complex disassembly. GO terms for cellular compartment and molecular function showed that ribosomal proteins were overrepresented. Given the generation of autophagosomes from phagophores formed from the ER, it is plausible that a range of ER‐resident, ribosomal and membrane proteins are overrepresented in autophagosomes.

Previous work which investigated autophagosome isolation from HEK 293 cells, followed by 2D gel electrophoresis, in‐gel digestion and analysis by MALDI‐TOF mass spectrometry, identified 101 proteins [48]. Of this list of putative autophagosomal proteins, we also identified 23 proteins in our data of which 9 (Z‐VAD‐fmk) and 7 (NGLY1 KD) were found to be significantly enriched in our analyses (Fig. S7a,b). It should be noted that in addition to the different processing and analysis approaches, autophagy was induced by calcium phosphate precipitate in this previous study [48]. One further comparison was performed to a list of 94 autophagosomal proteins enriched from MCF‐7 cells which were determined to localize to autophagosomes independent of the trigger of cellular autophagy [49]. Here, a greater overlap of 37 out of the 94 proteins which were also identified in our study was observed. However, significant enrichment was seen for only 8 (Z‐VAD‐fmk) and 6 (NGLY1 KD) of these 37 proteins with most of the remainder of proteins identified, but found not to be significantly altered in our experiments (Fig. S8a,b). This suggests that the more comparable experimental approaches (analysis by LC‐MS/MS and control for FDR using target‐decoy database search approach) enabled a greater overlap of the data sets. However, other confounding factors, such as the expected biological differences between MCF‐7, as a cancer cell line, as opposed to the HEK 293 nonmalignant cells utilized in our studies, result in a number of quantitative differences in autophagosome formation and maturation. These proteomic investigations reveal similar autophagosomal protein composition following either Z‐VAD‐fmk treatment or NGLY1 KD in HEK 293 cells and extend our understanding of factors which are enriched in cellular autophagosomes following the triggering of cellular autophagy by impairment or inhibition of NGLY1.

In conclusion, we have shown that cellular treatment with the pan‐caspase inhibitor Z‐VAD‐fmk leads to an induction of autophagy which is mediated by the inhibition of NGLY1. No such increase in cellular autophagy is observed with the alternative caspase inhibitor Q‐VD‐OPh. The increase in GFP‐LC3 puncta per cell upon Z‐VAD‐fmk treatment or NGLY1 KD occurs due to an upregulation in autophagosome formation, and no disruption of autophagic flux can be observed. Isolation of autophagosomes by IP, following Z‐VAD‐fmk treatment or NGLY1 KD, and mass spectrometry‐based proteomics analysis identifies a similar range of autophagosomal proteins and protein cargo. Analysis of GO terms for the autophagosome‐enriched proteins highlights a number of overrepresented biological processes including protein translation, protein localization and targeting, mRNA degradation and protein complex disassembly as well as ribosomal complexes. Our results show clear evidence for a causal relationship between NGLY1 disruption and induction of cellular autophagy, a result which may have implications for novel treatment modalities for NGLY1 disorder. Our findings furthermore make a strong case for the preferential use of Q‐VD‐OPh for cellular studies of caspase inhibition where an off‐target‐mediated induction of autophagy is undesirable.

## Materials and methods

### Methylthiazolyl diphenyl tetrazolium bromide (MTT) cell viability assay

Cells were plated in a 96‐well plate (Greiner Bio One Ltd, Stonehouse, UK) at a density of 2000 cells per well. Cells were incubated with treatments as indicated per experiment. For WT MEF cell ATG13 siRNA knockdown, cells were transfected with 50 nm ATG13 siRNA or nontargeting siRNA (Thermo Fisher, Loughborough, UK, #AM16708) for 4 days prior to treatment. MEF cells were transfected using Lipofectamine RNAiMax as per manufacturer’s instructions (Thermo Fisher). Following treatment, media was aspirated and replaced with fresh media (100 µL) containing MTT (final concentration 0.2 mg·mL^−1^). Plates were incubated at 37 °C for 2 h. Following incubation, media containing MTT was carefully removed and DMSO (100 µL) was added to each well. The plates were then shaken for 20 min at RT. The absorbance was read using a BMG Labtech FLUOstar OPTIMA plate reader at 570 nm. Treatment with menadione (20 µm, 24 h) was included as a positive control to confirm decreased absorbance in nonviable cells.

### Quantification of levels of GFP‐LC3‐positive puncta

GFP‐LC3 HEK 293 cells were plated on uncoated 16‐mm glass coverslips at (40 000 cells per well) and treated with Z‐VAD‐fmk or Q‐VD‐OPh (50 µm, 24–72 h) or a vehicle control. Cells were imaged on the oil immersion 63× objective. A minimum of three areas per coverslip were imaged and were selected under bright field. The number of GFP‐LC3‐positive puncta was counted manually, and the average number of puncta per cell was calculated per condition. To measure flux, GFP‐LC3 HEK 293 cells were treated with Bafilomycin A1 (100 nm, 1 h) or 3‐MA (5 mm, 1 h) at 37 °C at 5% CO_2_. Cells were reimaged, and the number of GFP‐LC3 puncta was calculated.

### qPCR determination of ER stress markers

Assessment of ER stress markers was carried out by quantitative real‐time PCR (qPCR). qPCR was used to determine changes in ER stress markers and *N*‐glycanase downregulation for siRNA‐mediated knockdown. The sequences of all primers used are presented in Table S3. RT‐qPCR was carried out using an MJ Opticom real‐time PCR machine. For analysis of ER stress markers, cells were treated with Z‐VAD‐fmk or Q‐VD‐OPh (50 µm, 24–72 h) or a vehicle control and MG132 (5 µm, 18 h). RNA was extracted using RNeasy Mini Kit. RNA (100 ng) was used with the One‐Step Luna^®^ Universal qPCR Master Mix per manufacturer’s instructions. Samples were heated to 55 °C, 10 min to convert RNA to cDNA followed by an initial denaturation at 98 °C, 5 min followed by denaturation at 95 °C, 30 s, annealing at 58 °C, 30 s, extension at 72 °C, 30 s for 42 cycles. The threshold was taken over the global minimum over 10 standard deviations and analysed using the double delta Ct method. GAPDH was employed as a housekeeping gene, and an untreated control was used as the baseline.

### Propidium iodide exclusion and Annexin V staining

Cells were grown in DMEM and plated into 12‐well plate (Greiner). HEK 293 cells were treated with either Q‐VD‐OPh or Z‐VAD‐fmk (50 µm, 24–72 h) or a vehicle control (DMSO). Menadione (20 µm, 18 h) was used as a positive control for cell death and apoptosis induction. Samples were treated with trypsin to detach cells and washed three times in PBS. Cells were incubated with the Molecular Probes™ Dead Cell Apoptosis Kit with Annexin V FITC and PI, for flow cytometry. Cells were resuspended in 1× Annexin binding buffer (100 µL). FITC Annexin V (5 µL) and propidium iodide (PI) (1 µL, 100 µg·mL^−1^) were added for 15 min at RT. Samples were diluted with Annexin binding buffer (400 µL), and red/green signal was analysed using a TALI image‐based cytometer (Invitrogen, Paisley, UK). The percentage of Annexin V‐positive and PI‐negative cells were calculated, and cell number was recorded.

### Deglycosylation‐dependent Venus (ddVENUS) assay

HEK 293 cells plated in 12‐well plates (Greiner) at a density of 80 000 cells per well were transfected with ddVENUS plasmid DNA (2 µg per well) using JetPEI^®^ HTS DNA transfection reagent (Polyplus‐transfection^®^) in accordance with the manufacturer protocol. After 24 h, Z‐VAD‐fmk (0–300 µm) was added and the cells incubated for a further 24 h. For genetic ablation of *N*‐glycanase, HEK 293 cells were transfected with SMARTpool: ON‐TARGETplus NGLY1 siRNA (25 nm) or an ON‐TARGETplus nontargeting control (25 nm) using JetPRIME^®^ DNA and siRNA transfection reagent (Polyplus‐transfection^®^) as per the manufacturer instructions. Cells were analysed 3 days post‐transfection.

The proteasome inhibitor, MG132 (8 µm), was added for 6 h before the cells were trypsinized, collected by centrifugation and resuspended in HBSS (500 µL). Cells were kept on ice before measurement. Fluorescence intensity was measured by flow cytometry on a BD FACSCalibur (BD Biosciences, Wokingham, UK) and analysed using BD CellQuest™. A minimum of 10 000 cells were analysed for each condition. A nontransfected well was used as a control to determine background fluorescence. Cells were analysed using the FL1 filter at 400 V. The nontransfected cells were gated out of analysis, and the median fluorescence intensity of transfected cells was plotted.

### Quantitation of thioflavin T (ThT) fluorescence by flow cytometry

HEK cells were treated with either Q‐VD‐OPh or Z‐VAD‐fmk (50 µm, 24–72 h) or a vehicle control (DMSO). For genetic ablation, HEK cells were transfected with SMARTpool: ON‐TARGETplus NGLY1 siRNA or an ON‐TARGETplus nontargeting control (25 nm, 3–5 days). Cells were loaded with thioflavin T (5 µm, 30 m) at 37 °C in complete media. Cells were detached with trypsin and washed in HBSS three times by centrifugation. Cells were resuspended in HBSS (500 µL) and analysed using BD FACSCalibur on the FL1 channel (ex 488 em 530), at 440 V, and 10 000 cells were counted per condition.

### Intracellular calcium fluorescence imaging

GFP‐LC3 HEK 293 cells were plated on uncoated 16‐mm glass coverslips at 40 000 cells per well and treated with Z‐VAD‐fmk, Q‐VD‐OPh (50 μm, 24–72 h) or a vehicle control. For genetic ablation of *N*‐glycanase, HEK 293 cells were transfected with SMARTpool: ON‐TARGETplus NGLY1 siRNA (25 nm) or an ON‐TARGETplus nontargeting control siRNA (25 nm) and analysed 3–5 days post‐transfection. Cells were incubated with FURA‐2 AM (1 μm) in imaging buffer (121 mm NaCl, 5.4 mm KCl, 0.8 mm MgCl_2_, 1.8 mm CaCl_2_, 6 mm NaHCO_3_, 5.5 mm D‐glucose, 25 mm HEPES, pH 7.4 supplemented with Gibco MEM amino acid and MEM nonessential amino acid solution) for 20 min at 37 °C. Cells were washed three times with imaging buffer and incubated for a further 20 min. Coverslips were imaged using a Leica DMI6000 fluorescence microscope on the 20× objective. Ca^2+^ was mobilized from intracellular stores using thapsigargin (1 μm) after 180 s. Images were taken every 20 s for 12 min. Time lapse videos were analysed using imagej [50]. Cytosolic areas of cells were selected plus a background region for 340 and 380 nm. A minimum of 20 cellular regions were analysed per coverslip. The 340/380 ratio was calculated. The area under the curve, baseline average of the first eight images and peak height were calculated using GraphPad Prism 7 (GraphPad Software, La Jolla, CA, USA, www.graphpad.com).

### Label‐free proteomics analysis

GFP‐LC3 HEK 293 cells were cultured as described above and treated with Z‐VAD‐fmk (50 µm) for 72 h or transfected with NGLY1 siRNA (25 nm) and used 5 days post‐transfection. Cells (80 million per replicate) were treated with bafilomycin A1 (100 nm) for 1 h to allow accumulation of autophagosomes. Following treatment with bafilomycin, cells were washed twice with ice‐cold PBS and harvested by treatment with 0.25% trypsin‐EDTA (Thermo Fisher). Cells were pelleted and washed three times in ice‐cold PBS. Cells were lysed in 1% Triton in PBS by Dounce homogenization and incubated for 0.5 h at 4 °C. The resultant lysate was centrifuged at 6000 ***g*** for 10 min at 4 °C. The supernatant was removed and centrifuged again at 20 000 ***g*** for 20 min at 4 °C. The supernatant was discarded and the pellet resuspended in PBS (500 µL) and added to filter spin columns (Corning) with GFPTrap^®^ agarose beads (50 µL) (ChromoTek GmbH, Munich, Germany) or control agarose beads (50 µL) (ChromoTek) and then washed three times in PBS. Samples were incubated with the agarose beads and tumbled for 1 h at 4 °C. The spin filter columns were centrifuged at 2500 ***g*** for 2 min, and the flow‐through discarded. Agarose beads were washed 7× in ice‐cold PBS‐T by centrifugation. Samples were eluted with 0.2 m glycine, pH 2.5 (50 µL) for 1 min with constant vortexing. Beads were centrifuged into a fresh, protein Lo‐Bind tubes (Eppendorf Ltd, Stevenage, UK). Elution was repeated, and the eluates were pooled. Samples were neutralized by the addition of 1 m phosphate buffer (pH 7.4). Eluted material was then processed for proteomic analysis by filter‐aided sample preparation (FASP) [51]. Tryptic digests were desalted and then analysed by liquid chromatography‐tandem mass spectrometry (LC‐MS/MS). Raw data were processed in the MaxQuant software utilizing the MaxLFQ label‐free quantification algorithm. Processed data were further analysed using the Perseus computational platform. After removal of known contaminant proteins and requiring quantification in at least two out of three biological replicates in at least one group, a total of 915 protein hits were obtained. Missing values were imputed (replacement of missing values from normal distribution; width 0.5, downshift 1.4) as described previously [52]. Protein enrichment and depletion were visualized using volcano plots and heatmap with hierarchical clustering analysis (HCA). Principal component analysis (PCA) was used to visualize protein abundance relationships across all samples. A schematic of the workflow is presented in Fig. S6a.

## Conflict of interest

The authors declare no conflict of interest.

### Author contributions

SHN contributed to methodology, involved in formal analysis, investigated the study, curated the data and wrote – original draft. MDB supervised and wrote – review and editing. HBK supervised, wrote – review and editing, and contributed to mass spectrometry and proteomic data analysis. JEG provided resources and wrote – review and editing. SAA conceptualized, supervised and wrote – original draft.

### Peer Review

The peer review history for this article is available at https://publons.com/publon/10.1111/febs.16345.

## Supporting information


**Fig. S1.** Z‐VAD‐fmk is not at or below toxic under 100 µM.
**Fig. S2.** Z‐VAD‐fmk reduced the ability of autophagy deficient cells to reduce MTT.
**Fig. S3.** Z‐VAD‐fmk causes transient increase in ThT fluorescence as measured by flow cytometry.
**Fig. S4.** Z‐VAD‐fmk or Q‐VD‐OPh treatment does not affect Ca^2+^ handling.
**Fig. S5.** NGLY1 siRNA knockdown does not affect Ca^2+^ handling.
**Fig. S6.** Schematic representation of proteomics analysis workflow.
**Fig. S7.** Comparison to: Gao, W., et al.*, Biochemical isolation and characterization of the tubulovesicular LC3‐positive autophagosomal compartment*. Journal of Biological Chemistry, 2010. 285(2): p. 1371‐1383.
**Fig. S8.** Comparison to: Dengjel, J., et al., *Identification of autophagosome‐associated proteins and regulators by quantitative proteomic analysis and genetic screens*. Molecular and Cellular Proteomics, 2012. 11(3):M111.014035.
**Table S1.** Gene ontology enrichment analysis ‐ hierarchical clustering analysis (HCA) cluster I – protein hits enriched in autophagosome IPs.
**Table S2.** Gene ontology enrichment analysis ‐ hierarchical clustering analysis (HCA) cluster II – protein hits enriched in negative control IPs.
**Table S3.** qPCR primer sequences.

## Data Availability

The mass spectrometry proteomic data have been deposited to the ProteomeXchange Consortium via the PRIDE partner repository [52] with the data set identifier PXD020367.

## References

[1] Schotte P , Declercq W , Van Huffel S , Vandenabeele P , Beyaert R . Non‐specific effects of methyl ketone peptide inhibitors of caspases. FEBS Lett. 1999;442:117–21. 9923616 10.1016/s0014-5793(98)01640-8

[2] Waterhouse NJ , Finucane DM , Green DR , Elce JS , Kumar S , Alnemri ES , et al. Calpain activation is upstream of caspases in radiation‐induced apoptosis. Cell Death Differ. 1998;5:1051–61. 9894612 10.1038/sj.cdd.4400425

[3] Misaghi S , Pacold ME , Blom D , Ploegh HL , Korbel GA . Using a small molecule inhibitor of peptide: N‐glycanaseto probe its role in glycoprotein turnover. Chem Biol. 2004;11:1677–87. 15610852 10.1016/j.chembiol.2004.11.010

[4] Deszcz L , Seipelt J , Vassilieva E , Roetzer A , Kuechler E . Antiviral activity of caspase inhibitors: effect on picornaviral 2A proteinase. FEBS Lett. 2004;560:51–5. 14987997 10.1016/S0014-5793(04)00069-9

[5] Garcia‐Calvo M , Peterson EP , Leiting B , Ruel R , Nicholson DW , Thornberry NA . Inhibition of human caspases by peptide‐based and macromolecular inhibitors. J Biol Chem. 1998;273:32608–13. 9829999 10.1074/jbc.273.49.32608

[6] Caserta TM , Smith AN , Gultice AD , Reedy MA , Brown TL . Q‐VD‐OPh, a broad spectrum caspase inhibitor with potent antiapoptotic properties. Apoptosis. 2003;8:345–52. 12815277 10.1023/a:1024116916932

[7] Chauvier D , Ankri S , Charriaut‐Marlangue C , Casimir R , Jacotot E . Broad‐spectrum caspase inhibitors: from myth to reality? [5]. Cell Death Differ. 2007;14:387–91. 17008913 10.1038/sj.cdd.4402044

[8] Kadigamuwa C , Choksi S , Xu Q , Cataisson C , Greenbaum SS , Yuspa SH , et al. Role of retinoic acid receptor‐γ in DNA damage‐induced necroptosis. iScience. 2019;17:74–86. 31255985 10.1016/j.isci.2019.06.019 PMC6606929

[9] Shindo R , Ohmuraya M , Komazawa‐Sakon S , Miyake S , Deguchi Y , Yamazaki S , et al. Necroptosis of intestinal epithelial cells induces type 3 innate lymphoid cell‐dependent lethal ileitis. iScience. 2019;15:536–51. 31132747 10.1016/j.isci.2019.05.011 PMC6538961

[10] Xu S , Huo J , Huang Y , Aw M , Chen S , Mak S , et al. von Hippel‐Lindau protein maintains metabolic balance to regulate the survival of naive B lymphocytes. iScience. 2019;17:379–92. 31351078 10.1016/j.isci.2019.07.002 PMC6660606

[11] Chen SY , Chiu LY , Maa MC , Wang JS , Chien CL , Lin WW . zVAD‐induced autophagic cell death requires c‐Src‐dependent ERK and JNK activation and reactive oxygen species generation. Autophagy. 2011;7:217–28. 21127402 10.4161/auto.7.2.14212 PMC3039770

[12] Wu YT , Tan HL , Huang Q , Sun XJ , Zhu X , Shen HM . ZVAD‐induced necroptosis in L929 cells depends on autocrine production of TNFα mediated by the PKC‐MAPKs‐AP‐1 pathway. Cell Death Differ. 2011;18:26–37. 20539307 10.1038/cdd.2010.72 PMC3131876

[13] Eichhold TH , Hookfin EB , Taiwo YO , De B , Wehmeyer KR . Isolation and quantification of fluoroacetate in rat tissues, following dosing of 2‐Phe‐Ala‐CH2‐F, a peptidyl fluoromethyl ketone protease inhibitor. J Pharm Biomed Anal. 1997;16:459–67. 9589405 10.1016/s0731-7085(97)00102-7

[14] Morrison JF , Peters RA . Biochemistry of fluoroacetate poisoning: the effect of fluorocitrate on purified aconitase. Biochem J. 1954;58:473–9. 13208639 10.1042/bj0580473 PMC1269923

[15] Suzuki T , Huang C , Fujihira H . The cytoplasmic peptide: N‐glycanase (NGLY1) – structure, expression and cellular functions. Gene. 2016;577:1–7. 26611529 10.1016/j.gene.2015.11.021 PMC4691572

[16] Suzuki T , Seko A , Kitajima K , Inoue Y , Inoue S . Identification of Peptide:N‐glycanase activity in mammalian‐derived cultured cells. Biochem Biophys Res Comm. 1993;194:1124–30. 8352768 10.1006/bbrc.1993.1938

[17] Grotzke JE , Lu Q , Cresswell P . Deglycosylation‐dependent fluorescent proteins provide unique tools for the study of ER‐associated degradation. Proc Natl Acad Sci USA. 2013;110:3393–8. 23401531 10.1073/pnas.1300328110 PMC3587246

[18] Suzuki T , Huang C , Harada Y , Hosomi A , Masahara‐Negishi Y , Seino J , et al. Endo‐β‐n‐acetylglucosaminidase forms N‐GlcNAc protein aggregates during ER‐associated degradation in NGLY1‐defective cells. Proc Natl Acad Sci USA. 2015;112:1398–403. 25605922 10.1073/pnas.1414593112 PMC4321286

[19] Enns GM , Shashi V , Bainbridge M , Gambello MJ , Zahir FR , Bast T , et al. Mutations in NGLY1 cause an inherited disorder of the endoplasmic reticulum‐associated degradation pathway. Genet Med. 2014;16:751–8. 24651605 10.1038/gim.2014.22 PMC4243708

[20] He P , Grotzke JE , Ng BG , Gunel M , Jafar‐Nejad H , Cresswell P , et al. A congenital disorder of deglycosylation: biochemical characterization of N‐glycanase 1 deficiency in patient fibroblasts. Glycobiology. 2015;25:836–44. 25900930 10.1093/glycob/cwv024 PMC4487302

[21] Tambe MA , Ng BG , Freeze HH . N‐glycanase 1 transcriptionally regulates aquaporins independent of its enzymatic activity. Cell Rep. 2019;29:4620–4631.e4. 31875565 10.1016/j.celrep.2019.11.097

[22] Lehrbach NJ , Breen PC , Ruvkun G . Protein sequence editing of SKN‐1A/Nrf1 by Peptide:N‐Glycanase controls proteasome gene expression. Cell. 2019;177:737–750.e15. 31002798 10.1016/j.cell.2019.03.035 PMC6574124

[23] Tomlin FM , Gerling‐Driessen UIM , Liu YC , Flynn RA , Vangala JR , Lentz CS , et al. Inhibition of NGLY1 inactivates the transcription factor Nrf1 and potentiates proteasome inhibitor cytotoxicity. ACS Central Science. 2017;3:1143–55. 29202016 10.1021/acscentsci.7b00224 PMC5704294

[24] Yang K , Huang R , Fujihira H , Suzuki T , Yan N . N‐glycanase NGLY1 regulates mitochondrial homeostasis and inflammation through NRF1. J Exp Med. 2018;215:2600–16. 30135079 10.1084/jem.20180783 PMC6170171

[25] Mueller WF , Jakob P , Sun H , Clauder‐Münster S , Ghidelli‐Disse S , Ordonez D , et al. Loss of N‐glycanase 1 alters transcriptional and translational regulation in K562 cell lines. G3: Genes ‐ Genomes ‐ Genetics. 2020;10:1585–97. 32265286 10.1534/g3.119.401031 PMC7202010

[26] Wu T , Li Y , Huang D , Han F , Zhang YY , Zhang DW , et al. Regulator of G‐protein signaling 19 (RGS19) and its partner Gα‐inhibiting activity polypeptide 3 (GNAI3) are required for zVAD‐induced autophagy and cell death in L929 cells. PLoS One. 2014;9:e94634. 24751948 10.1371/journal.pone.0094634 PMC3994006

[27] Kaizuka T , Mizushima N . Atg13 is essential for autophagy and cardiac development in mice. Mol Cell Biol. 2016;36:585–95. 26644405 10.1128/MCB.01005-15 PMC4751695

[28] Cox J , Mann M . MaxQuant enables high peptide identification rates, individualized p.p.b.‐range mass accuracies and proteome‐wide protein quantification. Nat Biotechnol. 2008;26:1367–72. 19029910 10.1038/nbt.1511

[29] Cox J , Hein MY , Luber CA , Paron I , Nagaraj N , Mann M . Accurate proteome‐wide label‐free quantification by delayed normalization and maximal peptide ratio extraction, termed MaxLFQ. Mol Cell Proteomics. 2014;13:2513–26. 24942700 10.1074/mcp.M113.031591 PMC4159666

[30] Tyanova S , Temu T , Sinitcyn P , Carlson A , Hein MY , Geiger T , et al. The Perseus computational platform for comprehensive analysis of (prote)omics data. Nat Methods. 2016;13:731–40. 27348712 10.1038/nmeth.3901

[31] Owings KG , Lowry JB , Bi Y , Might M , Chow CY . Transcriptome and functional analysis in a *Drosophila* model of NGLY1 deficiency provides insight into therapeutic approaches. Hum Mol Genet. 2018;27:1055–66. 29346549 10.1093/hmg/ddy026 PMC5886220

[32] Asahina M , Fujinawa R , Nakamura S , Yokoyama K , Tozawa R , Suzuki T . Ngly1 ‐/‐rats develop neurodegenerative phenotypes and pathological abnormalities in their peripheral and central nervous systems. Hum Mol Genet. 2020;29:1635–47. 32259258 10.1093/hmg/ddaa059 PMC7322575

[33] Rodriguez TP , Mast JD , Hartl T , Lee T , Sand P , Perlstein EO . Defects in the neuroendocrine axis contribute to global development delay in a *Drosophila* model of NGLY1 deficiency. G3: Genes ‐ Genomes ‐ Genetics. 2018;8:2193–204. 29735526 10.1534/g3.118.300578 PMC6027897

[34] Fujihira H , Masahara‐Negishi Y , Tamura M , Huang C , Harada Y , Wakana S , et al. Lethality of mice bearing a knockout of the Ngly1‐gene is partially rescued by the additional deletion of the Engase gene. PLoS Genet. 2017;13:e1006696. 28426790 10.1371/journal.pgen.1006696 PMC5398483

[35] Maynard JC , Fujihira H , Dolgonos GE , Suzuki T , Burlingame AL . Cytosolic N‐GlcNAc proteins are formed by the action of endo‐β‐N‐acetylglucosaminidase. Biochem Biophys Res Comm. 2020;530:719–24. 32782141 10.1016/j.bbrc.2020.06.127 PMC7508226

[36] Kim RJ , Hah YS , Sung CM , Kang JR , Park HB . Do antioxidants inhibit oxidative‐stress‐induced autophagy of tenofibroblasts? J Orthop Res. 2014;32:937–43. 24644146 10.1002/jor.22608

[37] Luyten T , Welkenhuyzen K , Roest G , Kania E , Wang L , Bittremieux M , et al. Resveratrol‐induced autophagy is dependent on IP3Rs and on cytosolic Ca2 +. Biochim Biophys Acta Mol Cell Res. 2017;1864:947–56. 28254579 10.1016/j.bbamcr.2017.02.013

[38] Zhang J , Chiu J , Zhang H , Qi T , Tang Q , Ma K , et al. Autophagic cell death induced by resveratrol depends on the Ca2+/AMPK/mTOR pathway in A549 cells. Biochem Pharmacol. 2013;86:317–28. 23680031 10.1016/j.bcp.2013.05.003

[39] Kong J , Peng M , Ostrovsky J , Kwon YJ , Oretsky O , McCormick EM , et al. Mitochondrial function requires NGLY1. Mitochondrion. 2018;38:6–16. 28750948 10.1016/j.mito.2017.07.008 PMC6038697

[40] Kabeya Y , Mizushima N , Ueno T , Yamamoto A , Kirisako T , Noda T , et al. LC3, a mammalian homologue of yeast Apg8p, is localized in autophagosome membranes after processing. EMBO J. 2000;19:5720–8. 11060023 10.1093/emboj/19.21.5720 PMC305793

[41] Mann SS , Hammarback JA . Molecular characterization of light chain 3. A microtubule binding subunit of MAP1A and MAP1B. J Biol Chem. 1994;269:11492–7. 7908909

[42] Harrison B , Kraus M , Burch L , Stevens C , Craig A , Gordon‐Weeks P , et al. DAPK‐1 binding to a linear peptide motif in MAP1B stimulates autophagy and membrane blebbing. J Biol Chem. 2008;283:9999–10014. 18195017 10.1074/jbc.M706040200

[43] Pankiv S , Alemu EA , Brech A , Bruun JA , Lamark T , Øvervatn A , et al. FYCO1 is a Rab7 effector that binds to LC3 and PI3P to mediate microtubule plus end ‐ directed vesicle transport. J Cell Biol. 2010;188:253–69. 20100911 10.1083/jcb.200907015 PMC2812517

[44] Tang Z , Lin MG , Stowe TR , Chen S , Zhu M , Stearns T , et al. Autophagy promotes primary ciliogenesis by removing OFD1 from centriolar satellites. Nature. 2013;502:254–7. 24089205 10.1038/nature12606 PMC4075283

[45] Lee JY , Koga H , Kawaguchi Y , Tang W , Wong E , Gao YS , et al. HDAC6 controls autophagosome maturation essential for ubiquitin‐selective quality‐control autophagy. EMBO J. 2010;29:969–80. 20075865 10.1038/emboj.2009.405 PMC2837169

[46] Oehme I , Linke JP , Böck BC , Milde T , Lodrini M , Hartenstein B , et al. Histone deacetylase 10 promotes autophagy‐mediated cell survival. Proc Natl Acad Sci USA. 2013;110:E2592–601. 23801752 10.1073/pnas.1300113110 PMC3710791

[47] Bhutia SK , Kegelman TP , Das SK , Azab B , Su ZZ , Lee SG , et al. Astrocyte elevated gene‐1 induces protective autophagy. Proc Natl Acad Sci USA. 2010;107:22243–8. 21127263 10.1073/pnas.1009479107 PMC3009793

[48] Gao W , Kang JH , Liao Y , Ding WX , Gambotto AA , Watkins SC , et al. Biochemical isolation and characterization of the tubulovesicular LC3‐positive autophagosomal compartment. J Biol Chem. 2010;285:1371–83. 19910472 10.1074/jbc.M109.054197 PMC2801263

[49] Dengjel J , Høyer‐Hansen M , Nielsen MO , Eisenberg T , Harder LM , Schandorff S , et al. Identification of autophagosome‐associated proteins and regulators by quantitative proteomic analysis and genetic screens. Mol Cell Proteomics. 2012;11:1–17. 10.1074/mcp.M111.014035 PMC3316729 22311637

[50] Schneider CA , Rasband WS , Eliceiri KW . NIH image to ImageJ: 25 years of image analysis. Nat Methods. 2012;9:671–5. 10.1038/nmeth.2089 22930834 PMC5554542

[51] Wiśniewski JR , Zougman A , Nagaraj N , Mann M . Universal sample preparation method for proteome analysis. Nat Methods. 2009;6:359–62. 19377485 10.1038/nmeth.1322

[52] Keilhauer EC , Hein MY , Mann M . Accurate protein complex retrieval by affinity enrichment mass spectrometry (AE‐MS) rather than affinity purification mass spectrometry (AP‐MS). Mol Cell Proteomics. 2015;14:120–35. 25363814 10.1074/mcp.M114.041012 PMC4288248

[53] Perez‐Riverol Y , Csordas A , Bai J , Bernal‐Llinares M , Hewapathirana S , Kundu DJ , et al. The PRIDE database and related tools and resources in 2019: improving support for quantification data. Nucleic Acids Res. 2019;47:D442–50. 30395289 10.1093/nar/gky1106 PMC6323896

