# Supplementary material for: Off‐target inhibition of NGLY1 by the polycaspase inhibitor Z‐VAD‐fmk induces cellular autophagy
Source: FEBS J. 2022 Jan 18;289(11):3115–31. doi: 10.1111/febs.16345 (PMC9304259; doi:10.1111/febs.16345)
Supplement: Supplementary file 1 — Fig. S1. Z‐VAD‐fmk is not at or below toxic under 100 µM. Fig. S2. Z‐VAD‐fmk reduced the ability of autophagy deficient cells to reduce MTT. Fig. S3. Z‐VAD‐fmk causes transient increase in ThT fluorescence as measured by flow cytometry. Fig. S4. Z‐VAD‐fmk or Q‐VD‐OPh treatment does not affect Ca2+ handling. Fig. S5. NGLY1 siRNA knockdown does not affect Ca2+ handling. Fig. S6. Schematic representation of proteomics analysis workflow. Fig. S7. Comparison to: Gao, W., et al., Biochemical isolation and characterization of the tubulovesicular LC3‐positive autophagosomal compartment. Journal of Biological Chemistry, 2010. 285(2): p. 1371‐1383. Fig. S8. Comparison to: Dengjel, J., et al., Identification of autophagosome‐associated proteins and regulators by quantitative proteomic analysis and genetic screens. Molecular and Cellular Proteomics, 2012. 11(3):M111.014035. Table S1. Gene ontology enrichment analysis ‐ hierarchical clustering analysis (HCA) cluster I – protein hits enriched in autophagosome IPs. Table S2. Gene ontology enrichment analysis ‐ hierarchical clustering analysis (HCA) cluster II – protein hits enriched in negative control IPs. Table S3. qPCR primer sequences. [file FEBS-289-3115-s001.zip › febs16345-sup-0001-Supinfo.pdf]

FEBS PRESS
science publishing by scientists
WILEY
The FEBS Journal
www.febsjournal.org

# Off-target inhibition of NGLY1 by the polycaspase inhibitor Z-VAD-fmk induces cellular autophagy

Sarah H. Needs, Martin D. Bootman, Jeff E. Grotzke, Holger B. Kramer and Sarah A. Allman

DOI: 10.1111/febs.16345

**Supplementary information**

# Title: Off-target inhibition of NGLY1 by the poly-caspase inhibitor Z-VAD-fmk induces cellular autophagy

*Sarah H Needs[1, 2], Martin D Bootman[1], Jeff E Grotzke[3], Holger B Kramer[4,5], Sarah A Allman[1,2,6*]*

[1]School of Life, Health and Chemical Sciences, The Open University, Walton Hall, Milton Keynes, MK7 6AA, UK

[2]Reading School of Pharmacy, University of Reading, Whiteknights, Reading, RG6 6AD, UK

[3]Yale University School of Medicine, New Haven, CT 06520, USA

[4]Department of Physiology, Anatomy and Genetics, University of Oxford, Parks Road, Oxford, OX1 3PT, UK

[5]MRC London Institute of Medical Sciences, Hammersmith Hospital Campus, Du Cane Road, London, W12 0NN, UK

[6]Leicester School of Pharmacy, De Montfort University, The Gateway, Leicester, LE1 9BH, UK

*Corresponding author: s.a.allman@reading.ac.uk

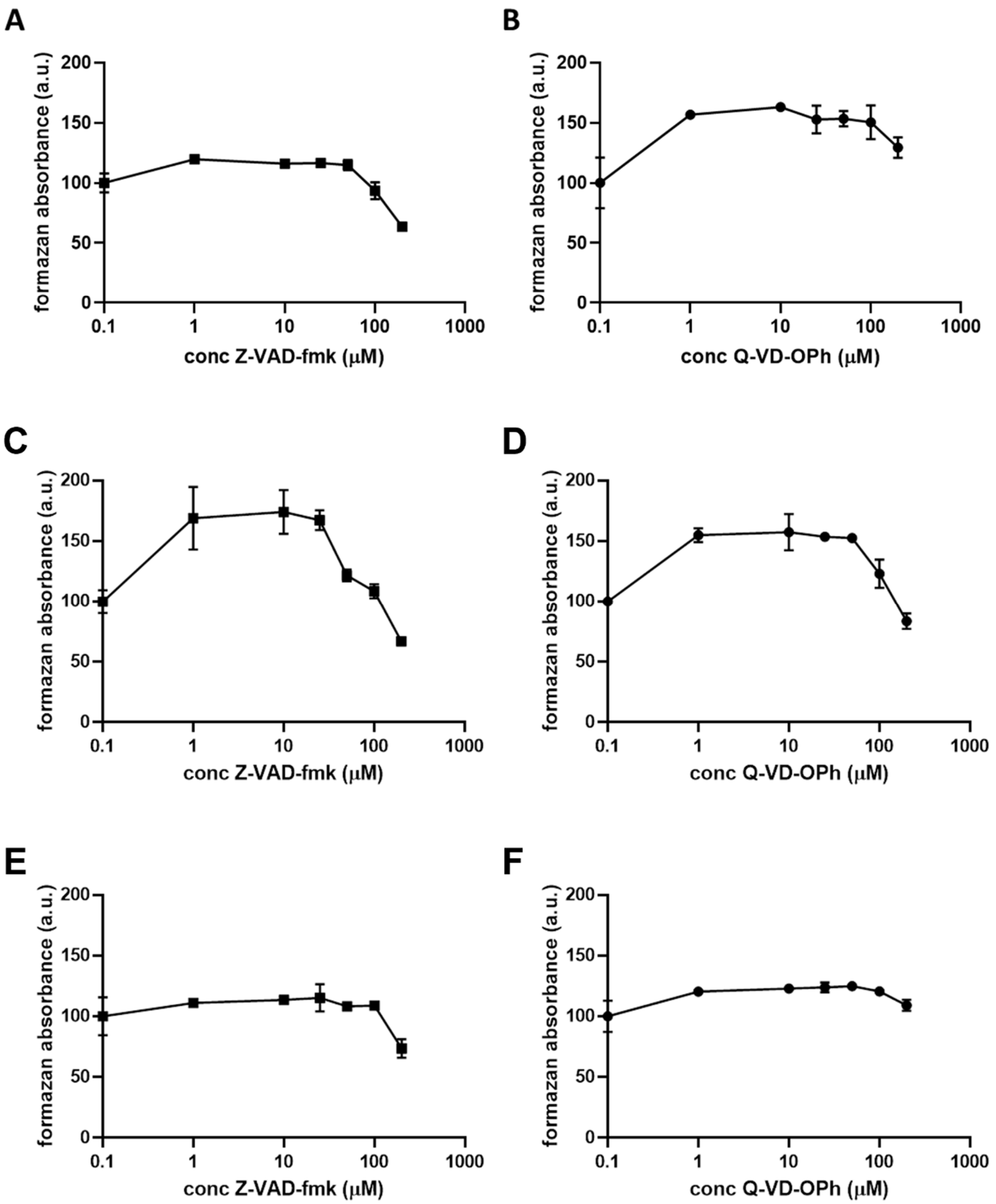


**Supplementary Figure S1. Z-VAD-fmk is not at or below toxic under 100 μM.** MTT assay of HEK 293 cells were treated with (**A**) Z-VAD-fmk (24 h) or (**B**) Q-VD-OPh (24 h), (**C**) Z-VAD-fmk (48 h) or (**D**) Q-VD-OPh (48 h) and (**E**) Z-VAD-fmk (72 h) or (**F**) Q-VD-OPh (72 h). Data was normalised to the vehicle control. Error bars ± SEM, n=3.

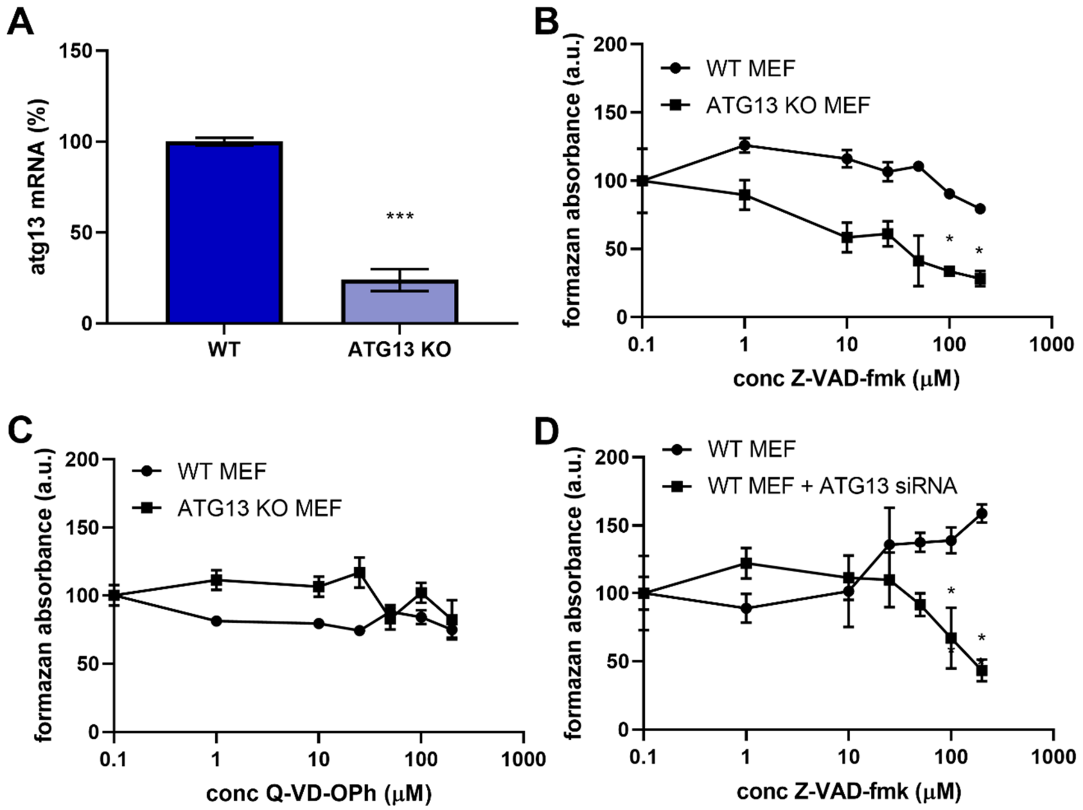


A**Supplementary Figure S2. Z-VAD-fmk reduced the ability of autophagy deficient cells to reduce MTT.** (**A**) MTT of ATG13 KO MEF and WT MEF treated with (**B**) Z-VAD-fmk (1-200 μM) or (**C**) Q-VD-OPh (1-200 μM) for 24 h. (**D**) MTT of WT MEF treated with ATG13 siRNA or non-targeting siRNA for 4 days followed by Z-VAD-fmk (1-200μM) for 24h. Multiple t-tests, Holm-Sidak. Error bars ± SEM, n=3, $p < 0.05$.

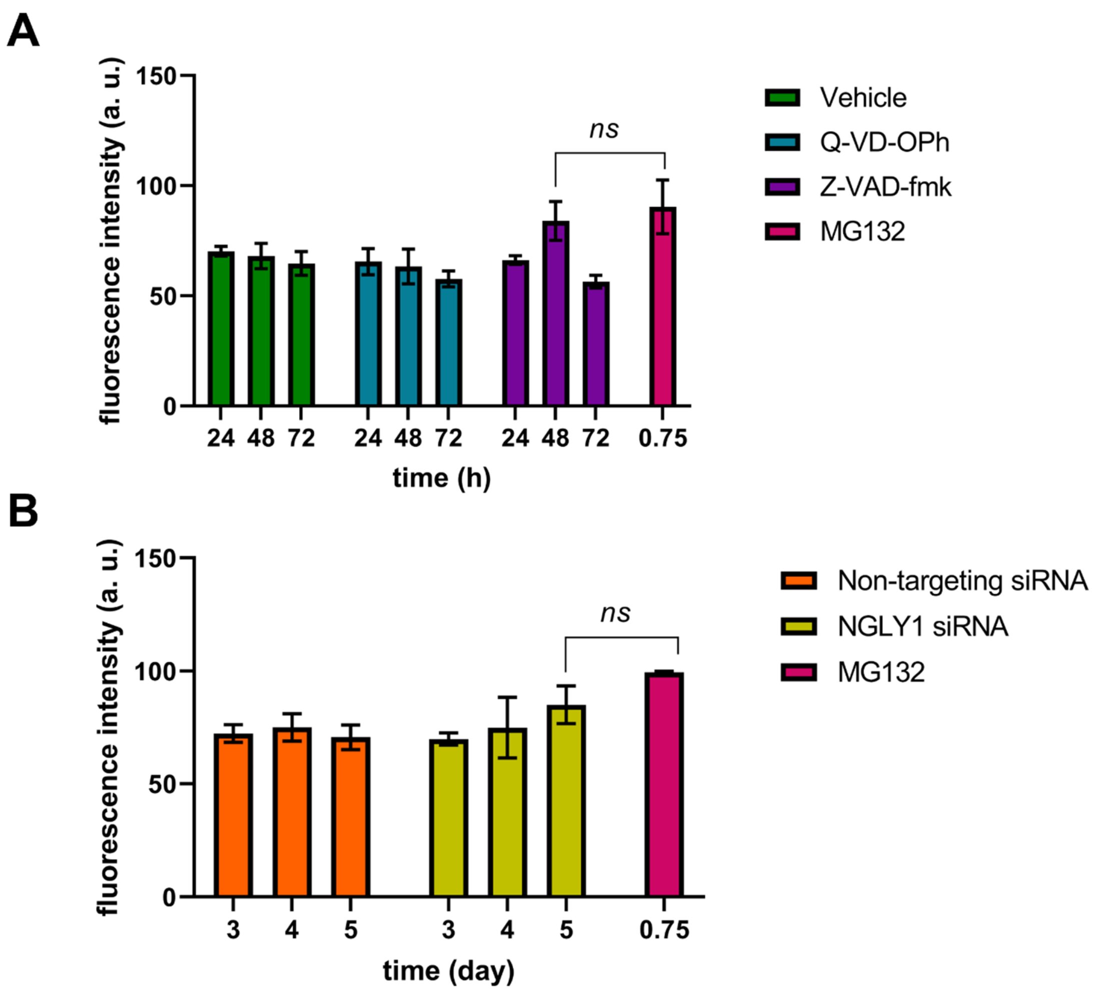


**Supplementary Figure S3 Z-VAD-fmk causes transient increase in ThT fluorescence as measured by flow cytometry.** (**A**) Flow cytometric analysis of HEK cells visualised with ThT after treatment with Z-VAD-fmk or Q-VD-OPh (50 µM, 24 – 72 h) or MG132 (100 nM). 10 000 cells were calculated per condition (n = 3). Two-way ANOVA, Tukey's post-hoc. Error bars ± SEM, n=3, $p < 0.05$. Significant differences are observed between MG132 (positive control) ($p < 0.05$) for all conditions (not marked) except for (**A**) 48 h post-Z-VAD-fmk treatment MG132 treatment and (**B**) 5 days post-NGLY1 siRNA KD where there is no significant difference between fluorescence intensity at these time points and the positive control (marked as *ns*).

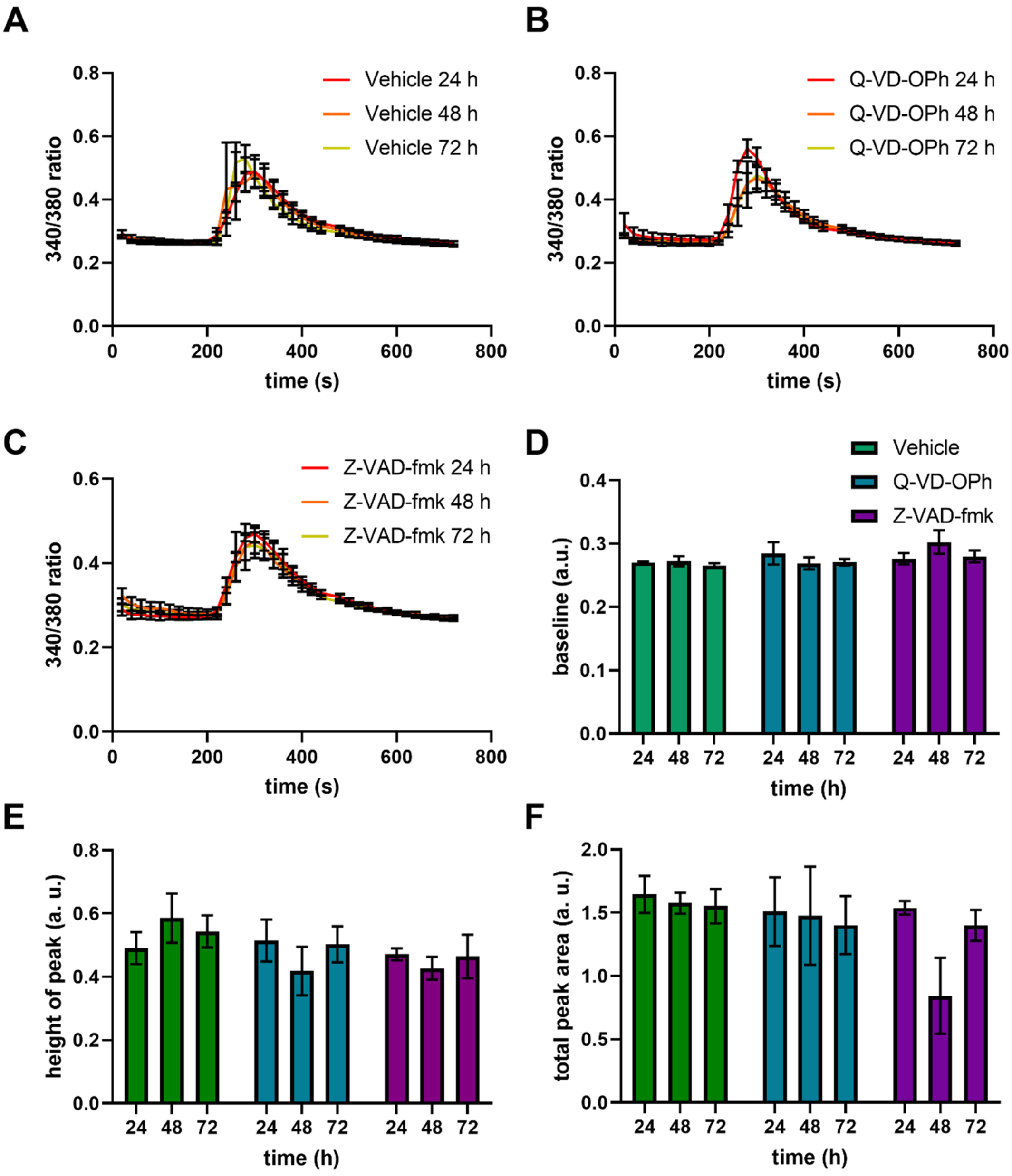


**Supplementary Figure S4. Z-VAD-fmk or Q-VD-OPh treatment does not affect $Ca^{2+}$ handling.** (**A-C**) 340/380 ratio traces of treated (vehicle, Q-VD-OPh or Z-VAD-fmk) HEK 293 cells loaded with Fura-2 AM and treated with thapsigargin (1 µM) at 180 s. (**D**) The baseline was averaged for the first 100 s (**E**) the maximum peak height was calculated and (**F**) area under the curve was determined. n=3, error bars indicate ± SEM.

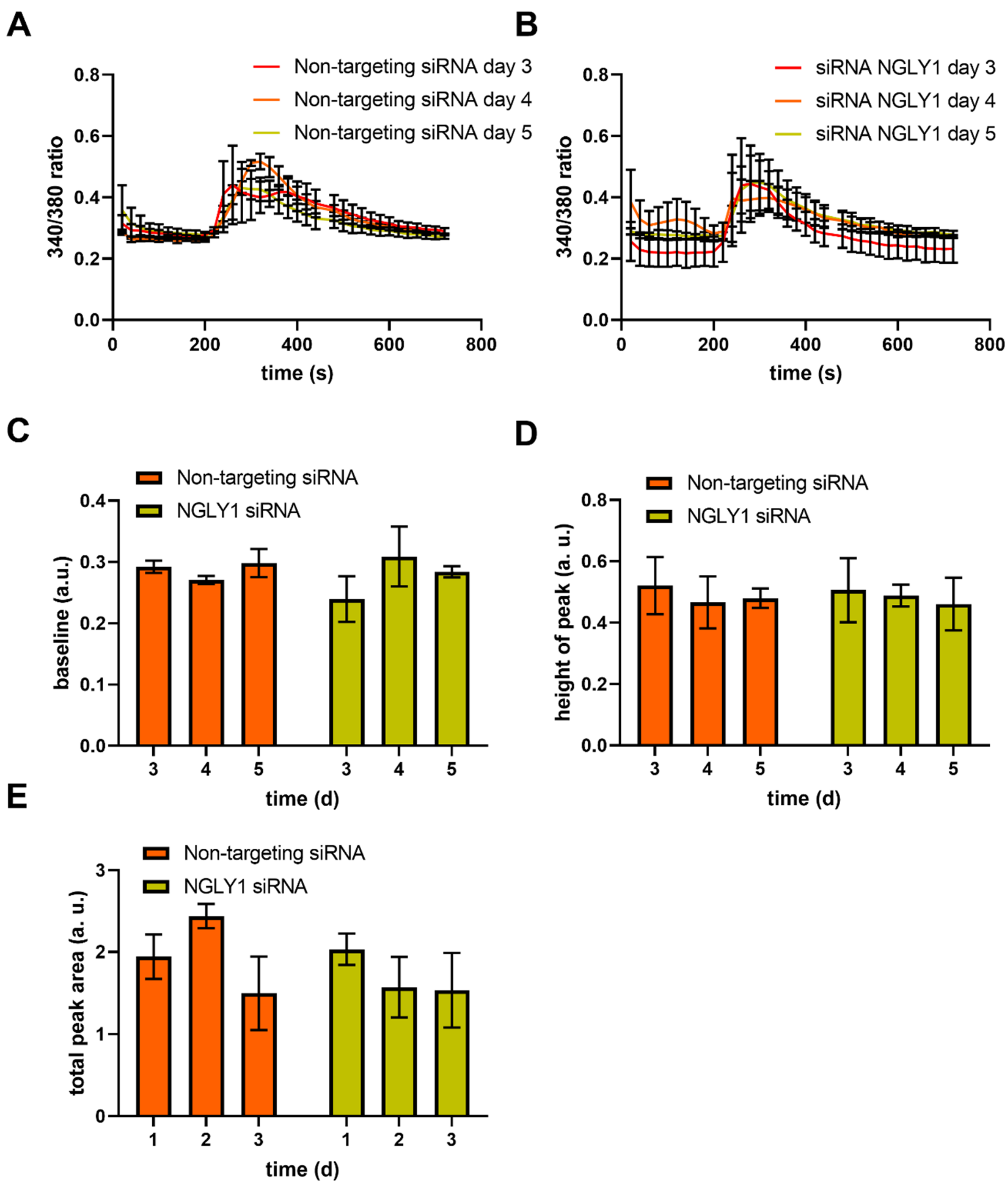


**Supplementary Figure S5. NGLY1 siRNA knockdown does not affect $Ca^{2+}$ handling.** (**A-B**) 340/380 ratio traces of HEK 293 cells (transfected with NGLY1 siRNA or non-targeting siRNA) loaded with Fura-2 AM and treated with thapsigargin (1 µM) at 180 s. (**C**) The baseline was averaged for the first 100 s, (D) the maximum peak height was calculated and (**E**) the area under the curve was taken. n=3, error bars indicate ± SEM.

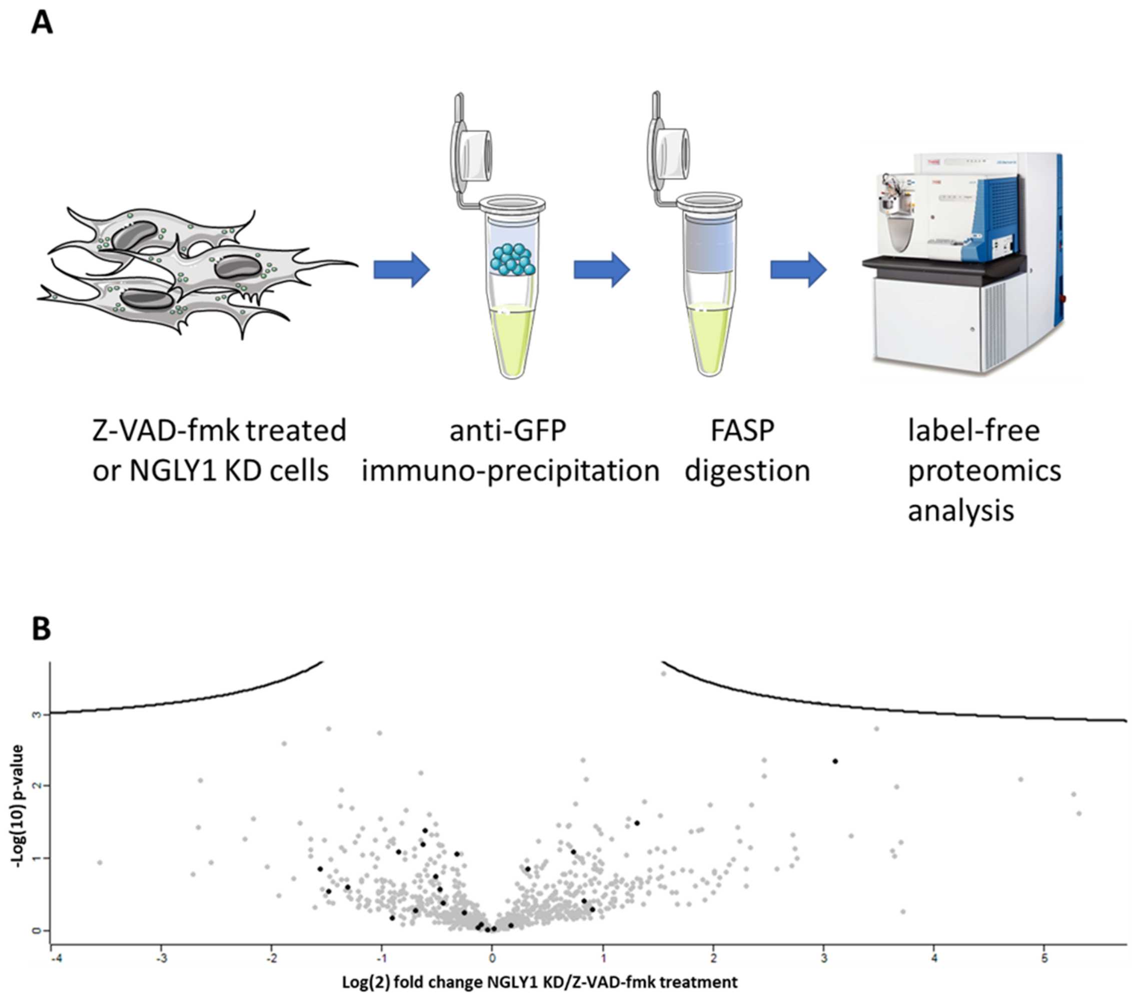


**Supplementary Figure S6. Schematic representation of proteomics analysis workflow. (A)** Schematic representation of workflow for label-free proteomics experiments (vector graphics adapted from Servier SMART Medical Art (https://smart.servier.com/) and used here under the terms of Creative Commons Attribution 3.0 Unported License **(B)** Label-free proteomics analysis of autophagosomes shows no significantly changing proteins between NGLY1 KD and Z-VAD-fmk treatment; Volcano plot (FDR: 0.05, s0: 0.1) showing autophagy-related protein hits not significantly altered (black).

Note that label free proteomics methods such as those employed in this study require enrichment rather than purification of the organelle to homogeneity.

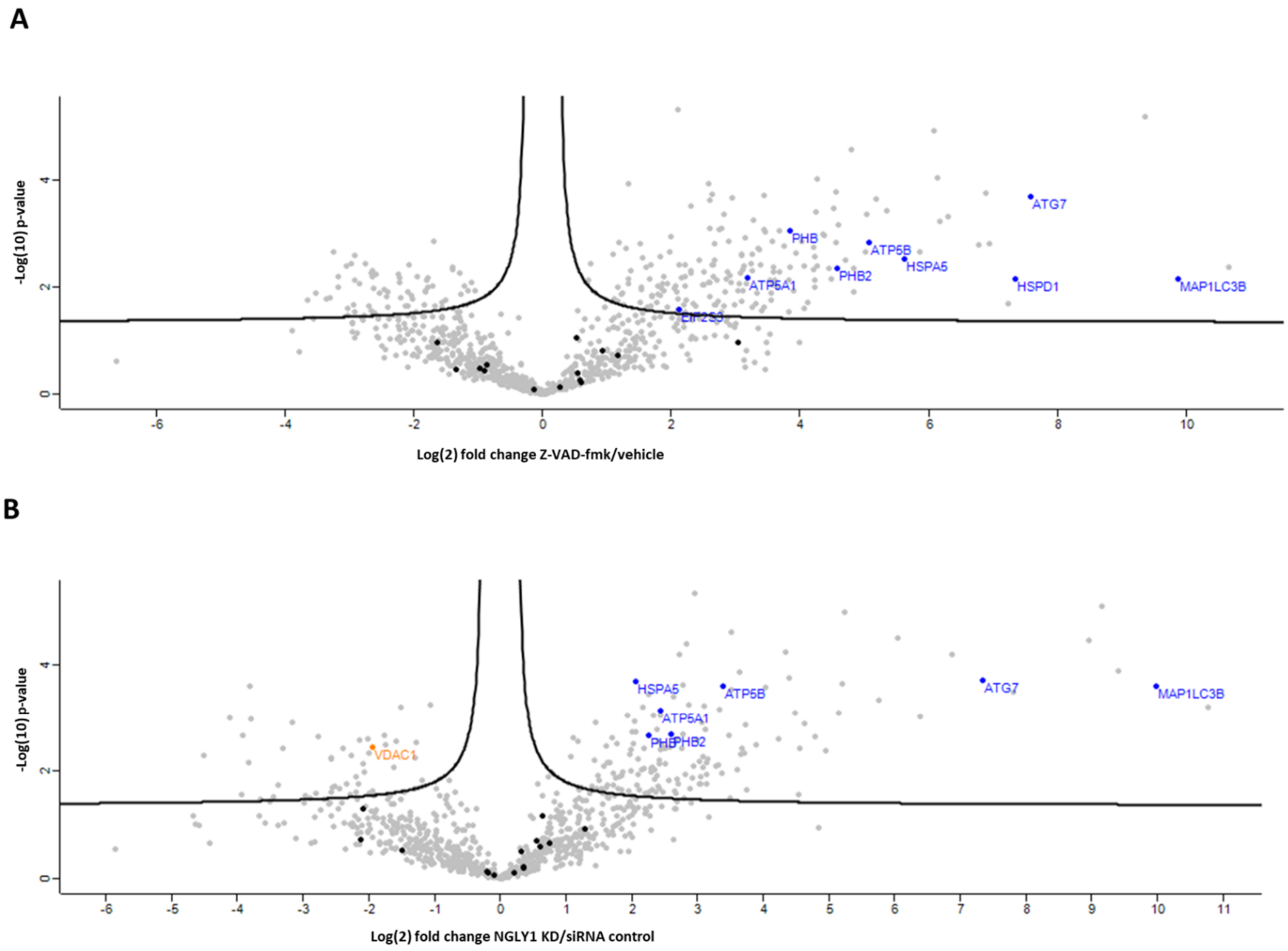


**Supplementary Figure S7. Comparison to: Gao, W., *et al., Biochemical isolation and characterization of the tubulovesicular LC3-positive autophagosomal compartment.* Journal of Biological Chemistry, 2010. 285(2): p. 1371-1383. (A)** Z-VAD-fmk treatment: 23 out of 101 proteins identified by Gao et al were identified in our study; of these 9

were significantly enriched (blue) and 14 were not significantly altered (black) **(B)** NGLY1 KD 23 out of 101 proteins identified by Gao et al were identified in our study; of these 7 were significantly enriched (blue), 1 was significantly depleted (orange) and 15 were not significantly altered (black); Volcano plot (FDR: 0.05, s0: 0.1).

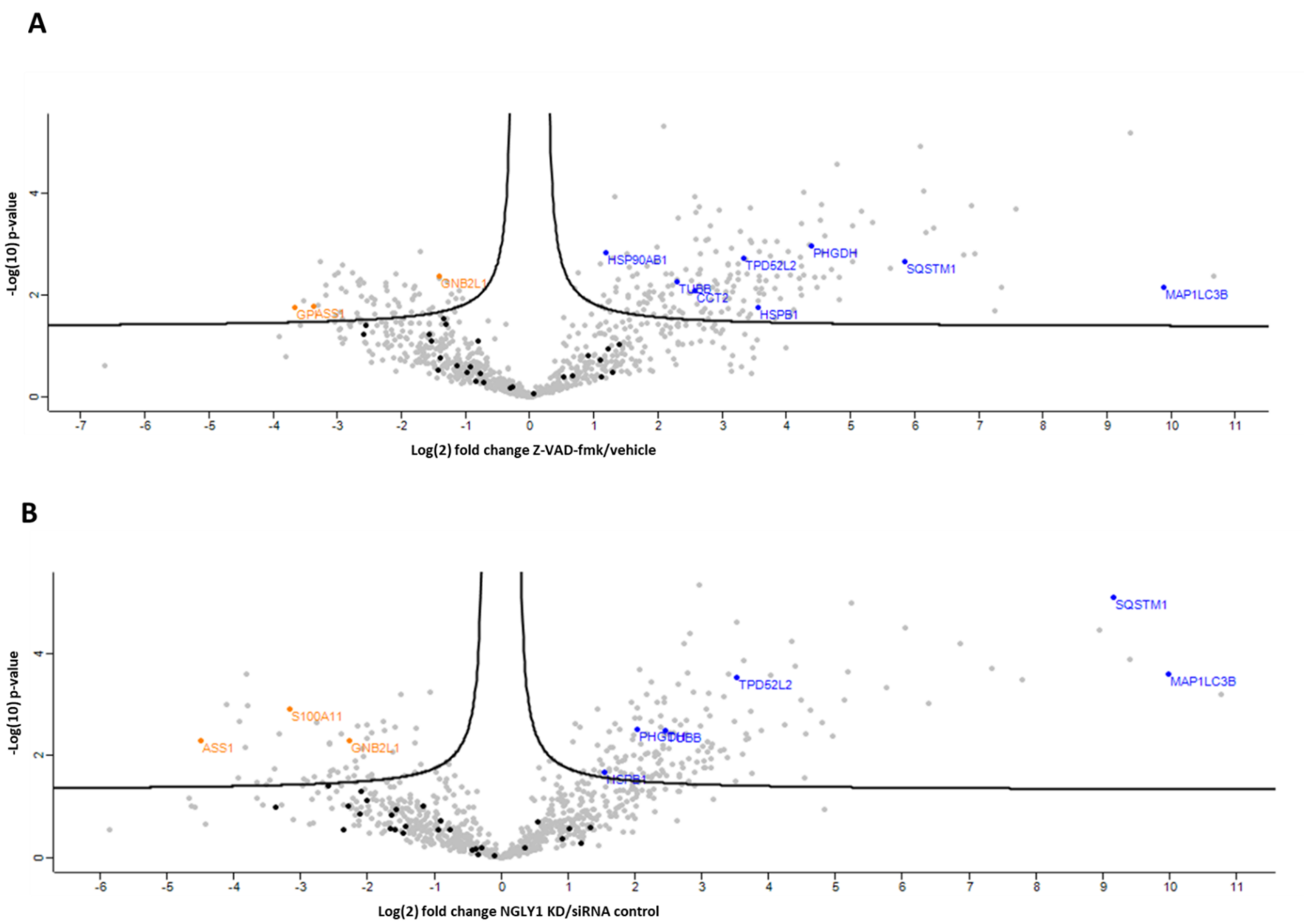


**Supplementary Figure S8. Comparison to: Dengjel, J., *et al*., *Identification of autophagosome-associated proteins and regulators by quantitative proteomic analysis and genetic screens.* Molecular and Cellular Proteomics, 2012. 11(3):M111.014035. (A)** Z-VAD-fmk treatment: 37 out of 94 proteins identified by Dengjel et al. were identified in our study; of these 8 were significantly enriched (blue), 3 were significantly depleted (orange) and 26 were not significantly altered (black) **(B)** NGLY1 KD 37 out of 94

proteins identified by Dengjel et al. were identified in our analysis; of these 6 were significantly enriched (blue), 3 were significantly depleted (orange) and 28 were not significantly altered (black); Volcano plot (FDR: 0.05, s0: 0.1).

**Table S1. Gene ontology enrichment analysis - hierarchical clustering analysis (HCA) cluster I – protein hits enriched in autophagosome IPs** Significantly overrepresented (Benjamini-Hochberg FDR <0.05) Gene Ontology (GO) terms for biological processes (GOBP), cellular compartment (GOCC) and molecular function (GOMF); total number of protein hits in cluster I: 228

| Gene Ontology term Biological Process (GOBP) | P value | Enrichment | Total no of proteins | Proteins in cluster | Ben. Ho. FDR |
|---|---|---|---|---|---|
| SRP-dependent cotranslational protein targeting to membrane | 2.42E-09 | 1.5301 | 55 | 52 | 1.56E-06 |
| protein targeting to ER | 2.42E-09 | 1.5301 | 55 | 52 | 1.96E-06 |
| establishment of protein localization to organelle | 3.87E-09 | 1.49 | 63 | 58 | 2.08E-06 |
| establishment of protein localization in endoplasmic reticulum | 2.42E-09 | 1.5301 | 55 | 52 | 2.61E-06 |
| protein targeting to membrane | 8.45E-10 | 1.5332 | 57 | 54 | 2.73E-06 |
| nuclear-transcribed mRNA catabolic process, nonsense-mediated decay | 6.82E-09 | 1.5268 | 53 | 50 | 3.15E-06 |
| cotranslational protein targeting to membrane | 2.42E-09 | 1.5301 | 55 | 52 | 3.91E-06 |
| establishment of localization | 1.18E-08 | 1.2365 | 178 | 136 | 4.77E-06 |
| transport | 1.35E-08 | 1.2392 | 175 | 134 | 4.85E-06 |
| viral transcription | 4.02E-08 | 1.4985 | 54 | 50 | 1.30E-05 |
| viral infectious cycle | 4.58E-08 | 1.4789 | 58 | 53 | 1.35E-05 |
| intracellular transport | 7.97E-08 | 1.2716 | 140 | 110 | 1.98E-05 |
| viral reproductive process | 7.53E-08 | 1.4441 | 65 | 58 | 2.03E-05 |
| translational termination | 1.07E-07 | 1.4939 | 52 | 48 | 2.31E-05 |
| mRNA catabolic process | 1.20E-07 | 1.4739 | 56 | 51 | 2.42E-05 |
| establishment of localization in cell | 1.05E-07 | 1.2576 | 148 | 115 | 2.43E-05 |
| protein transport | 1.28E-07 | 1.2974 | 121 | 97 | 2.43E-05 |
| protein complex disassembly | 1.92E-07 | 1.4713 | 55 | 50 | 3.28E-05 |
| nuclear-transcribed mRNA catabolic process | 1.92E-07 | 1.4713 | 55 | 50 | 3.46E-05 |
| translational elongation | 3.08E-07 | 1.4686 | 54 | 49 | 4.53E-05 |
| cellular protein complex disassembly | 3.08E-07 | 1.4686 | 54 | 49 | 4.74E-05 |

| | | | | | |
|---|---|---|---|---|---|
| establishment of protein localization | 2.95E-07 | 1.2818 | 125 | 99 | 4.78E-05 |
| RNA catabolic process | 4.94E-07 | 1.4481 | 57 | 51 | 6.66E-05 |
| macromolecular complex disassembly | 4.94E-07 | 1.4481 | 57 | 51 | 6.95E-05 |
| translation | 5.81E-07 | 1.4042 | 68 | 59 | 7.51E-05 |
| cellular macromolecular complex disassembly | 7.78E-07 | 1.445 | 56 | 50 | 9.68E-05 |
| intracellular protein transport | 1.91E-06 | 1.2947 | 105 | 84 | 0.00022886 |
| cellular process involved in reproduction | 2.66E-06 | 1.3804 | 68 | 58 | 0.00030736 |
| translational initiation | 3.71E-06 | 1.4026 | 60 | 52 | 0.00041379 |
| cellular component disassembly | 4.01E-06 | 1.3769 | 67 | 57 | 0.00043213 |
| mRNA metabolic process | 4.54E-06 | 1.3452 | 77 | 64 | 0.00047341 |
| macromolecule catabolic process | 5.79E-06 | 1.3524 | 73 | 61 | 0.00058497 |
| cellular component disassembly at cellular level | 6.01E-06 | 1.3732 | 66 | 56 | 0.00058875 |
| cellular macromolecule catabolic process | 7.26E-06 | 1.3604 | 69 | 58 | 0.00069101 |
| reproductive process | 1.53E-05 | 1.2947 | 90 | 72 | 0.0014119 |
| protein targeting | 2.55E-05 | 1.3111 | 79 | 64 | 0.0022884 |
| cellular component organization or biogenesis at cellular level | 3.85E-05 | 1.1678 | 176 | 127 | 0.0031965 |
| cellular component organization at cellular level | 3.85E-05 | 1.1678 | 176 | 127 | 0.0032807 |
| cellular macromolecular complex subunit organization | 5.85E-05 | 1.2869 | 83 | 66 | 0.0047311 |
| protein complex subunit organization | 0.0001632 | 1.2674 | 83 | 65 | 0.012875 |
| cellular protein metabolic process | 0.0003578 | 1.1689 | 144 | 104 | 0.027558 |

| **Gene Ontology term Cellular Compartment (GOCC)** | **P value** | **Enrichment** | **Total no of proteins** | **Proteins in cluster** | **Ben. Ho. FDR** |
|---|---|---|---|---|---|
| large ribosomal subunit | 7.10E-07 | 1.6184 | 28 | 28 | 0.00019796 |
| cytosolic large ribosomal subunit | 7.10E-07 | 1.6184 | 28 | 28 | 0.00039591 |
| ribonucleoprotein complex | 6.12E-05 | 1.2797 | 86 | 68 | 0.011382 |
| macromolecular complex | 0.00034 | 1.1169 | 213 | 147 | 0.031636 |

| **Gene Ontology term Molecular Function (GOMF)** | **P value** | **Enrichment** | **Total no of proteins** | **Proteins in cluster** | **Ben. Ho. FDR** |
|---|---|---|---|---|---|
| structural constituent of ribosome | 3.15E-08 | 1.5213 | 50 | 47 | 2.33E-05 |
| structural molecule activity | 8.55E-06 | 1.3487 | 72 | 60 | 0.0031628 |

**Table S2. Gene ontology enrichment analysis - hierarchical clustering analysis (HCA) cluster II – protein hits enriched in negative control IPs** Significantly overrepresented (Benjamini-Hochberg FDR <0.05) Gene Ontology (GO) terms for biological processes (GOBP), cellular compartment (GOCC) and molecular function (GOMF); total number of protein hits in cluster II: 141

| Gene Ontology term Biological Process (GOBP) | P value | Enrichment | Total no of proteins | Proteins in cluster | Ben. Ho. FDR |
|---|---|---|---|---|---|
| response to inorganic substance | 3.08E-05 | 2.2038 | 19 | 16 | 0.0026967 |

| Gene Ontology term Cellular Compartment (GOCC) | P value | Enrichment | Total no of proteins | Proteins in cluster | Ben. Ho. FDR |
|---|---|---|---|---|---|
| extracellular region | 0.000151 | 2.3989 | 12 | 11 | 0.021062 |
| extracellular space | 0.000189 | 1.8319 | 30 | 21 | 0.021063 |

**Table S3. qPCR primer sequences**

| Species | Gene | Sequence |
|---|---|---|
| Human | GAPDH Forward | 5’- AGGGCTGCTTTTAACTCTGGT-3’ |
| | GAPDH Reverse | 5’-CCCCACTTGATTTTGGAGGGA-3’ |
| | BiP (Grp78) Forward | 5’-ACGTGGAATGACCCGTCTGT-3’ |
| | BiP (Grp78) Reverse | 5’-AACCACCTTGAACGGCAAGA-3’ |
| | CHOP Forward | 5’-ACCAAGGGAGAACCAGGAAACG-3’ |
| | CHOP Reverse | 5’-TCACCATTCGGTCAATCAGAGC-3’ |
| | NGLY1 Forward | 5’-GGTTTGAAGCTCGCTATGTTTGGGATTAC-3’ |
| | NGLY1 Reverse | 5’-CTTGTCACAGACATCTTCACATGCATCAC-3’ |
| Mouse | ATG13 Forward | 5’- AGGGCGGGAGAGATCGTTTG-3’ |
| | ATG13 Reverse | 5’- CAGCACAGGTCGCAGAGAGA-3’ |
